# Interparticle Communication and Lithium Dynamics in Faceted Nickel-Rich NMC Cathodes

**DOI:** 10.1021/jacs.5c15171

**Published:** 2026-01-21

**Authors:** Veronika Šedajová, Gabriela Horwitz, Jiho Han, Alice J. Merryweather, George S. Phillips, Vikram S. Deshpande, Norman A. Fleck, Akshay Rao, Clare P. Grey

**Affiliations:** † Yusuf Hamied Department of Chemistry, 2152 University of Cambridge, Lensfield Road, CB2 1EW Cambridge, U.K.; ‡ The Faraday Institution, Quad One, Harwell Science and Innovation Campus, OX11 0RA Didcot, U.K.; § Cavendish Laboratory, University of Cambridge, J.J. Thomson Avenue, CB3 0US Cambridge, U.K.; ∥ Department of Engineering, 2152 University of Cambridge, Trumpington Street, Cambridge CB2 1PZ, U.K.; ⊥ Illumion Ltd., Maxwell Centre, J.J. Thomson Avenue, Cambridge CB3 0HE, U.K.

## Abstract

Lithium-ion batteries have transformed energy storage solutions with the layered materials LiNi_*x*_Mn_*y*_Co_*z*_O_2_ (NMC) at the forefront of commercial cathodes due to their superior performance. However, understanding the complex dynamics of lithium-ion diffusion within these materials remains a challenge, as conventional models used to analyze experimental results often simplify particle size distributions, intercalation kinetics and mechanisms, often ignoring interactions between particles. This study explores the anisotropic lithium-ion transport in NMC cathodes with octahedral particle morphologies. Using charge photometry (CP) under charge-rest protocols, the research unveils distinct transport behaviors across different facets of the particle. These findings, corroborated through finite element simulations, highlight the critical role of interparticle communication—an interaction previously underappreciated but shown here to be significant. By combining experimental evidence with computational modeling, this study provides a deeper comprehension of lithium-ion transport and the underlying mechanisms, offering valuable insights to future studies of active materials for battery applications.

## Graphical abstract

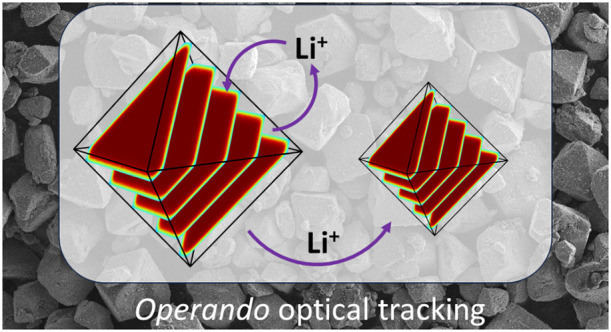

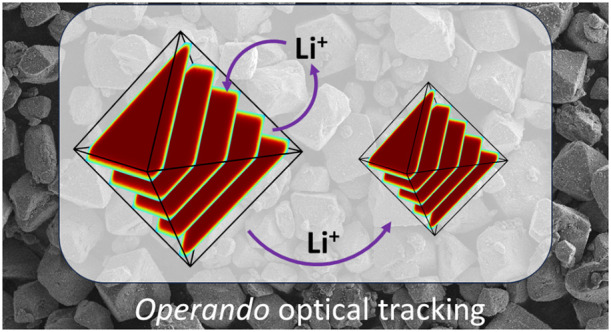

## 1 Introduction

While lithium-ion batteries have revolutionized energy storage technologies, improvements to the technology are continuously emerging from research in the fields of materials chemistry and engineering. In this context, layered cathodes (positive electrode materials), such as the family LiNi_*x*_Mn_*y*_Co_*z*_O_2_ (NMC), now represent state-of-the-art commercial cathode materials with high energy density and good cyclability.^1,2^

Traditional NMC cathodes are composed of polycrystalline spherical secondary particles that are formed of many smaller single-crystalline primary particles. These polycrystalline materials suffer from intergranular particle cracking between the different primary particles upon cycling, resulting in a loss of capacity;^3–8^ hence, in recent years, there has been a shift to the use of larger single-crystalline materials.^9–11^ In this second type of material, each particle is ideally a single crystal with distinct crystallographic facets. The basal plane (001), parallel to the layered structure of lithium and transition metal oxides’ layers, is Li^+^-impermeable, while nonbasal planes allow for Li^+^ (de)­intercalation during electrochemical processes. Hence, (de)­lithiation occurs anisotropically from the faces that dissect the (0001) planes comprising the alternating crystalline Li–O and TM–O layers (TM = transition metal).^12^ The primary particles within the larger secondary polycrystalline materials are generally randomly oriented (although we note that synthesis methods, particularly those of Sun and co-workers, have been developed to orient the primary particles^13^) and it is reasonable to assume close-to-spherical diffusion into the secondary particles; however, when it comes to the single-crystal materials, the diffusion is anisotropic, and clear differences are expected between different facets.^14,15^

Considerable effort has been devoted to understanding the lithiation dynamics of intercalation materials in battery-relevant configurations, and many models have been developed to understand and predict their behavior. However, most of these models assume a unimodal and narrow particle size distribution of spheres and a uniform local current density at the surface of the particles.^16–21^ Particular interest lies in the accurate description of the transport of Li^+^ within the solid, which, if slow, contributes to transport overpotentials, irreversible capacity loss, and poor rate capabilities.^22–25^ At a particle level, Grey and co-workers have shown, using models with state of change (SOC)-dependent diffusivity, that the core–shell behavior of (de)­lithiation leads to the first cycle irreversible capacity loss.^12^ Chueh and co-workers have recently analyzed the population behavior of differently sized particles and shown the effect of limiting intercalation kinetics on the lithiation of NMC particles. They concluded that the smaller particles experience a preferential acceleration of the delithiation process.^26^ This originates from the intercalation resistance dependence on SOC, it being higher near fully delithiated or lithiated states. Consequently, as the smaller particles have a larger surface-to-volume ratio, they are delithiated faster than the larger particles on charging; they then experience less resistance to the deintercalation reaction, facilitating further delithiation. Population dynamics become increasingly important considering the recently developed strategies for electrode engineering^27^ involving the use of bimodal particle size distributions^28,29^ or blending of different cathode chemistries.^30–32^

Here, we explore the behavior of distinct crystallographic planes in a widely used cathode active material, LiNi_0.84_Mn_0.07_Co_0.09_O_2_ (“NMC811”), by employing the recently developed charge photometry (CP),^33–35^ to obtain valuable insights into lithium-ion diffusion via recording an optical response during battery operation. The investigated material contains octahedral morphology single-crystalline particles, which allows us to distinguish between the basal ((0001), inactive) and side (exposed layers, active) facets and track their activity in the same experiment. The crystallographic facets display differences in optical behavior, highlighting the anisotropic nature of Li^+^ ion movement within the crystals. The specially designed optical coin cell (Figure S1b) allows operation under standard battery testing protocols (charging, discharging, voltage hold, and resting period) with concurrent optical imaging. The intensity of backscattered and reflected light is largely governed by the local dielectric properties of the particle, which exhibit sensitivity to variations in the lithiation state, as previously reported in an NMC-like material.^34,36^

To understand the distinct optical behavior of different crystallographic planes and the role of particle size further, this study examines the behavior of NMC particles and electrodes under charge-rest experiments, mimicking the conditions of galvanostatic intermittent titration techniques (GITTs).^37,38^ Experimental observations are complemented with finite element simulations, offering a comprehensive explanation of the experimentally observed trends.^12^ We demonstrate the role of interparticle communication during charge-rest protocols, where the Li^+^ is shown to equilibrate between particles via the electrolyte during the rest steps. The effect of particle size distribution is explored, in combination with kinetic and diffusion limitations on the lithiation behavior. These findings underscore the need to account for collective particle interactions in interpreting and modeling Li^+^ ion diffusion and transport mechanisms in battery systems.

## 2 Materials and Methods

### 2.1 Materials, Electrode and Cell Preparation

The active, octahedron-shaped NMC cathode material with nominal composition: LiNi_0.84_Mn_0.07_Co_0.09_O_2_ (Figure 1a,b), named OCT in this work,and used in the experimental part of this work was kindly supplied by Umicore N.V. This work used an optical coin-cell format.

**Figure 1:**
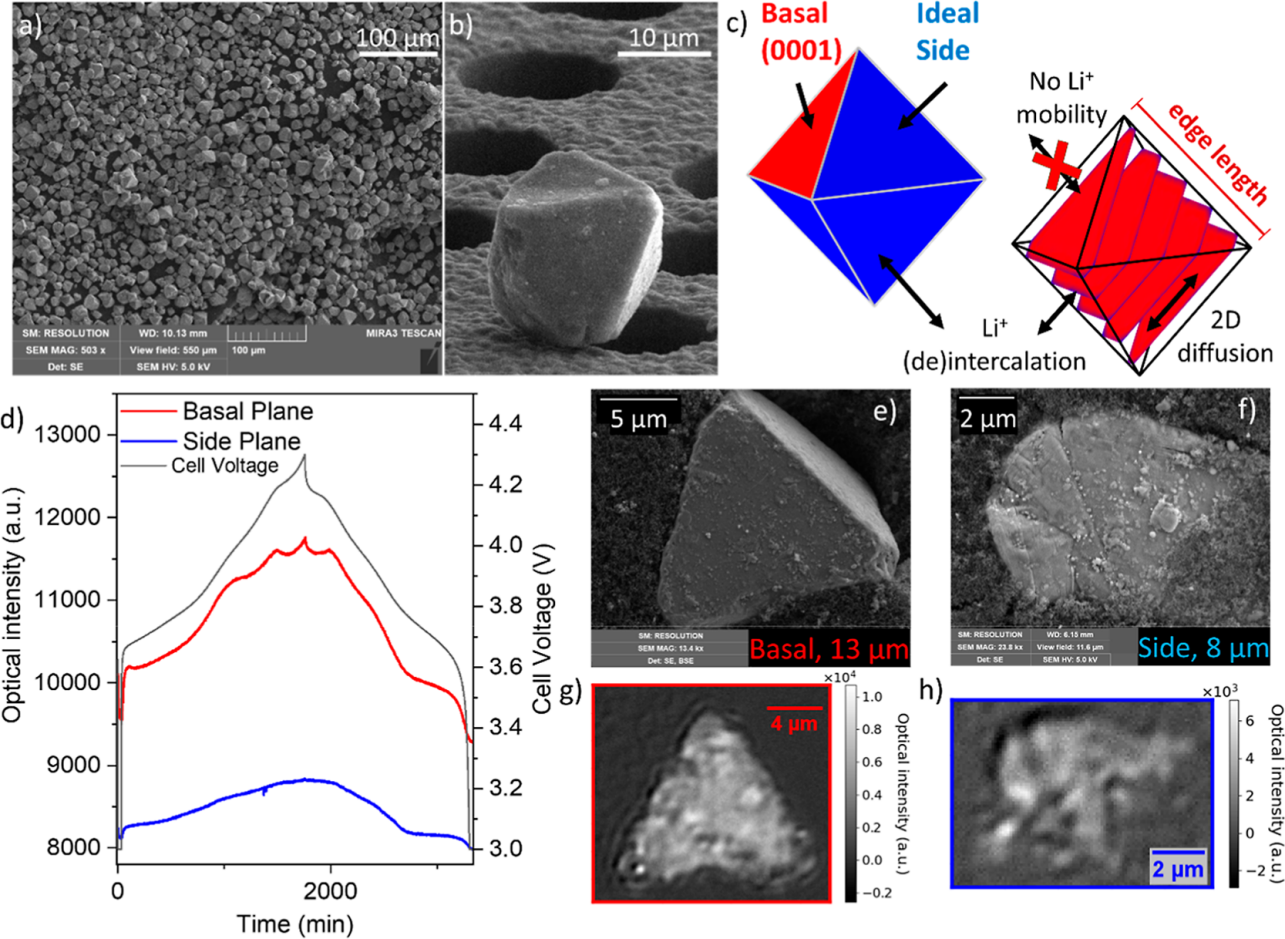
Particle morphology and initial optical behavior. (a) SEM image of OCT particles loosely placed on a Si grid, with (b) zoomed-in image of a single particle in a more dilute region. (c) Schematic representations of the idealized structure for the octahedral particles. Both representations are equivalent and presented in the same orientation, showing the exposed planes on the surface (left) and the crystallographic ab layers (right). (d) Optical intensity curves of two different facets (red and blue, corresponding to a basal and a side plane, respectively) as a function of time and cell voltage (black) during the C/30 rate cycle between 3.0 and 4.3 V. The presented intensity values correspond to the integrated intensity over the whole facet of the particle. A C/3 discharge to 3 V and a hold of 30 min at 3 V was performed before the C/30 cycle. (e,f) SEM images of two optically imaged particles in an electrode, post-mortem, embedded in the carbon matrix, alongside (g,h) their optical images, as typical representatives of a basal and a side plane. These example optical images show the raw intensity changes between the 632nd (start of the charging, with 1 min C/3 cycles with 10 min rests) and 1000th frame, calculated by subtracting the former from the latter.

Self-standing electrodes for optical coin-cell testing were prepared in an Ar-filled glovebox using the NMC active material, OCT, polytetrafluoroethylene (PTFE, previously dried at 80 °C under vacuum) binder, and conductive additive SuperP (TimCal, previously dried at 120 °C under vacuum) in a mass ratio of 70:20:10. OCT powder and SuperP were first mixed using a Thinky mixer at 2000 rpm for 10 min in a sealed container. Then, PTFE powder was added, and all three powders were mixed at 2000 rpm for another 10 min. The mixture was ground using a mortar and pestle until it formed self-standing film pieces, which were then pressed and rolled with a 150 μm slit rolling pin. If a homogeneous film could not be formed by using only the dry ingredients, a few drops of *N*-methyl-2-pyrrolidone were added. This produced a homogeneous and self-standing thin-film electrode, which was dried at 120 °C under vacuum overnight. All operations apart from mixing using the Thinky mixer were performed in the glovebox to prevent moisture contamination.

All coin cells were assembled with an OCT-containing electrode as the cathode, one lithium metal disk as the anode, one piece of GF-B separator, and the needed amount of the LP57 electrolyte (1 M LiPF_6_ in ethylene carbonate (EC)/ethyl methyl carbonate (EMC) 3:7 vol %): 160 μL for optical coin cells. All coin cells were assembled in an Ar-filled glovebox. The optical coin cell comprises of a circular glass microscopy coverslip, glued to the top casing of a regular coin cell with a 9 mm diameter hole. Al mesh (Cambridge Energy Solutions) is used as a current collector, placed next to the separator (the schematic can be seen in Figure S1b).

### 2.2 Electrochemical Measurements (Cycling and Charge-Rest Experiments)

The assembled coin cells were tested by using standard galvanostatic cycling (constant current charge and discharge), typically using a potential window of 3.0–4.3 V. Various rates were used throughout this study, where the *C*-rate was calculated by assuming a practical capacity of 220 mAh g^–1^. Due to the larger size of the particles and cell design, voltage holds at 3.0 V of different durations were applied after discharge or at the start of the experiments.

Charge-rest experiments were performed by using constant current pulses of varying lengths at different *C*-rates, followed by varying times of rest, depending on the type of experiment. The first current pulse has its time normalized, starting at 0 s.

Operando electrochemical and optical measurements were performed concurrently, as described below, using a PalmSens EmStat4S HR, controlled by a custom Python-based GUI.

### 2.3 Charge Photometry

CP was conducted using a wider field-of-view adaptation of a previously reported microscope setup,^34^ more specifically, a custom-built inverted wide-field microscope fitted with an air objective (60×/NA0.95 CFI Plan Apochromat Lambda 60XC, Nikon) with wide-field illumination (740 nm, Thorlabs fiber-coupled LED, M740F2). Polarization optics imaged the sample onto a CMOS camera (FLIR, Grashopper3 GigE, GS3-PGE-91S6M-C). The overall magnification was 60× with a full field of view of 188 μm × 118 μm (98 nm per pixel) limited by the camera sensor area. An optically accessible coin cell allowed for imaging of the active material in the self-standing electrode while applying standard battery cycling procedures. The optical coin cell was mounted with a custom cell holder onto an inverted microscope stage (Mad City Laboratories, MicroStage and Nano ZL500). The sample focus position was actively controlled, as described in Merryweather et al.^34^ The microscopy and electrochemistry were controlled concurrently by using a custom Python-based interface. We note that this microscope setup formed a prototype for a charge photometry instrument (″illumionONE”) that has subsequently been commercially developed by the company “Illumion”.

While the optical intensity is known to qualitatively reflect the local lithiation state in layered oxides,^34,39^ establishing a fully quantitative correlation for our material (LiNi_0.84_Mn_0.07_Co_0.09_O_2_) under operando conditions is challenging. The optical response is highly sensitive to experimental factors, including the objective depth, electrolyte thickness, and local particle morphology. As such, a universal calibration curve would require slow cycling (e.g., C/100) of individual particles under highly controlled conditions, which was found to be highly impractical for the present study, mainly due to optical drift and the size of the obtained data. Instead, our analysis focuses on relative intensity variations within individual particles, which reliably capture the lithiation dynamics, as validated by internal consistency across multiple experiments.

### 2.4 Characterization Techniques

Scanning electron microscopes, TESCAN MIRA3 FEG-SEM, TESCAN CLARA 2, and Helios 600i, were used to assess the morphology of the particles before and after cycling. All particle tracking was performed after electrochemical and optical measurements. Unfortunately, the 40 μm thick electrode (Figure S11) was severely damaged during the decrimping process of the optical coin cell, making it unsuitable for post-mortem SEM tracking.

### 2.5 Finite Element Simulations: Governing Equations and Implementation

Numerical simulations of the diffusion process of delithiation were carried out with a three-dimensional (3D) model of the octahedral particle geometries with the basal planes oriented as shown in the diagram in Figure 1c.

The governing equations were described in a work by Pandurangi et al.^40^ and a longer description of their derivation can be found in the Supporting Information in Note 1. Briefly, we define the lithiation state, or lithium occupancy fraction, θ­(*x*,*t*), as

1 $$\theta \left(x,t\right)=\frac{N_{Li^{+}}\left(x,t\right)}{N_{tot}}$$

where *N*_Li_(*x*,*t*) is the molar density (i.e., moles of Li in NMC volume unit) at a given position and time while *N*_tot_ is the total number of lattice sites available for Li occupancy, derived from X-ray diffraction (XRD) data and equivalent to 49,200 mol m^–3^.^40^

First, we assume that all cathode particles are identical in shape and size so that they all respond in the same manner. Subsequently, we assume that the particles differ and undergo individual rates of lithiation and delithiation, depending upon their shapes and sizes. The NMC particles are treated as a nonideal mixture of NMC and lithium, where the driving force for diffusion depends on the occupancy-dependent chemical potential of Li in the NMC lattice, μ_Li^+^_^NMC^, related to the open circuit voltage, *V*_oc_(θ) vs Li^+^/Li^0^ via equation

2 $$\mu_{Li^{+}}^{NMC}=-FV_{oc}\left(\theta \right)+\mu_{Li^{+}}^{a}+F\phi^{NMC}$$

where *F* is the Faraday constant, μ_Li^+^_^a^ is the chemical potential of Li in the anode, and ϕ^NMC^ is the electric potential in the cathode.

Then, the Li^+^ flux within the solid cathode, **J**, is driven by the gradient in chemical potential modulated by the diffusion coefficient, *D*, which can be described by the nonideal Fickian relation

3 $$J=-\frac{DN_{Li}}{RT}\cdot \nabla \mu_{Li^{+}}^{NMC}$$

The fact that μ_Li^+^_^a^ is constant, and by assuming the spatial gradient of ϕ^c^ is negligible (supported by the good electronic conductivity of NMC811), we arrive at the expression

4 $$J=\frac{FN_{tot}}{RT}\theta D\cdot \frac{\partial \left(V_{oc}\right)}{\partial \theta }\nabla \theta$$

The change in the Li concentration with time, *t*, is then given by

5 $$\frac{\partial N_{Li^{+}}}{\partial t}=-\nabla \cdot J$$

NMC is composed of a layered crystal structure, where, in the absence of antisite defects, diffusion occurs only in a transversely isotropic manner within the layers, i.e., parallel to the *ab*-plane (0001) of the crystallographic structure.^41^ Hence, neglecting the effect of antisite disordering, the diffusion is treated as isotropic within the *ab*-plane, and the diffusion tensor in the crystallographic basis takes the form

6 $$D=\left[\begin{array}{lll} D_{ab} & 0 & 0\\ 0 & D_{ab} & 0\\ 0 & 0 & 0 \end{array}\right]$$

For the simpler case of a distribution of identical particles, the ionic flux **J** into a representative particle of active surface, **S**, and volume *V* is given by

$$\int_{S}J\cdot n\;dS=C\frac{\rho VQ}{3600F}$$ 7

where ρ is the density of NMC, taken to be 4.78 g cm^–3^,^42^ and *Q* is the nominal capacity of 220 mAh g^–1^. The *C*-rate, *C*, is defined as the ratio of current to the battery’s nominal capacity in units of 1/h, hence the factor of 3600 in the denominator to convert hours to seconds.

Furthermore, given the anisotropic geometry of the simulated particles, the local ionic flux at each point on the surface of the cathode particles is nonuniform and depends on the lithiation kinetics at the electrolyte/NMC interface. These depend on the lithiation state and the overpotential η at the electrolyte/cathode interface, given by the Butler–Volmer equation

8 $$J\cdot n=\frac{j_{0}}{F}\left[e^{0.5F/RT\eta }-e^{-0.5F/RT\eta }\right]\;on\;S$$

with

9 $$\eta =\phi -V_{OC}\left(\theta \right)$$

Here, we make use of the fact that this is a one-electron reaction and assume that the charge transfer coefficient, α, equals 0.5. The exchange current density parameter, *j*_0,_ is defined by the current of the forward and back reactions when the system is at equilibrium. A fuller description of the terms that determine the overpotential, η, can be found in the Supporting Information.

We emphasize that voltage difference ϕ is spatially uniform throughout the cathode particles but *V*_OC_(θ) and consequently the overpotential, η, vary spatially over the surface of each cathode particle. Consequently, flux ***J*** also varies over the surface of the particle in accordance with the Butler–Volmer relation ([Disp-formula eq8]).

We then determined the lithiation state θ­(*x*, *t*) within each particle. The Butler–Volmer boundary condition (eq 8) is imposed for the surface flux, and the spatially uniform potential difference ϕ­(*t*) is set such that the prescribed galvanostatic *C*-rate is achieved.

A small modification is needed to the numerical approach for the case of multiple particles of varying shapes and sizes. Equation 7 is now modified such that **S** is the total surface of all particles, and the volume *V* is now the total volume of all particles. Hence, the lithiation state, θ­(*x*, *t*), and the cell potential, ϕ­(*t*), are numerically solved for. These equations were implemented in their weak form by introducing the virtual field θ̂(*x*,*t*) such that

10 $$\int_{\Omega }\left(N_{tot}\frac{\partial \theta }{\partial t}\mathrm{\theta ̂}-N_{tot}\theta D\cdot F\frac{\partial V_{oc}}{\partial \theta }\nabla \theta \cdot \nabla \mathrm{\theta ̂}\right)dV+\int_{S}\frac{j_{0}}{F}\left[e^{0.5F/RT\eta }-e^{-0.5F/RT\eta }\right]\mathrm{\theta ̂}dS=0$$

where Ω represents the volume of the simulated geometry and *S* is the active surface. The conversion of the equation system into its weak form is a standard method to relax the continuity requirements and simplify the numerical solution process. The steps to obtain this form from the original mass balance PDE system have already been described elsewhere.^40^

Equations 7–10 are implemented using the “Weak form PDE” module in COMSOL Multiphysics (v6.2). The 3D geometry of octahedral particles is oriented with their *ab*-planes (0001) parallel to the *X* and *Y* geometry frame of reference, and the system is solved in Cartesian coordinates.

The nonlinear behavior for the diffusion and intercalation kinetics was incorporated into the simulations by using experimental literature values for *D*(θ) and *j*_0_(θ). For *D*(θ), literature values were taken from Pandurangi et al. without further modification.^12,40^ For the exchange current density parameter, the normalized exchange current density, *j*_0_(θ)/*j*_0_(θ = 0.5) was extracted from Chueh et al.^26^ and rescaled to equate *j*_0_(θ = 0.5) = 0.28 mA/cm^2^ obtained from Kendrick et al.^43^ The values for open circuit voltage, *V*_OC_(θ), were taken from ref 6 and the particle was assumed to be fully lithiated when θ = 0.95.

The delithiation–diffusion processes of systems composed of more than one particle (two different-sized octahedral particles with edge sizes of 5 and 10 μm and 10-particle systems with different particle size distributions) were simulated in order to represent the smaller and larger particles seen experimentally. The main distinction in the multiparticle configuration simulations lies in the definition of the domains where the flux equations are applied. In these simulations, the flux across the *ab*-parallel surfaces (0001) of each particle was set to zero, while the flux across all active surfaces (side planes) was defined by a common set of eqs 7–9, with *S* corresponding to the ensemble of all side planes in the system. By imposing the flux conditions simultaneously on all active surfaces, without particle-specific differentiation, all particle surfaces were coupled to collectively satisfy the governing equations. By doing so, it is implied that the ionic transport in the electrolyte and electronic transport in the carbon-binder matrix occur freely and are not taken to be limiting. Charge-rest experiments were simulated, where the *C*-rate was set to 1 h^–1^ (1C) for the pulse steps, followed by relaxation periods of *C*-rate = 0 h^–1^. The integration in eq 7 was performed across the active surface of both particles, hence setting an overall current of the “sample”. This pulse-rest sequence was then repeated 10 times. Simulations were also performed for systems composed of 10 particles, where the edge sizes were generated according to different particle size distributions.

Moreover, the simulations assume smooth particle surfaces. While local roughness may influence local Li^+^ kinetics, its effect is expected to be secondary to the edge-related heterogeneities captured in the presented model and will be considered in future work. Additionally, electronic transport through the carbon–binder network is assumed to be nonlimiting. Thus, the observed phenomena arise primarily from electrochemical potential differences driven by particle size-dependent lithiation states.

## 3 Results and Discussion

### 3.1 SEM

SEM images of the LiNi_0.84_Mn_0.07_Co_0.09_O_2_ particles can be seen in Figure 1a,b, evidencing that the sample contains a range of octahedrally shaped particles but with different particle sizes, with a particle size distribution ranging from 5 to 30 μm, centered around 14 μm (Figures 1a and S1a). The idealized morphology of a single-crystal octahedron is shown in Figure 1c, while panel b shows a close to “perfect” experimental sample. The ideal OCT surface is dominated by the side (01–12) facets as they correspond to 6 out of a total of 8 facets of an octahedron and thus 75% of the total exposed surface. These facets are active for Li^+^ intercalation, as both the transition metal and Li^+^ layers are exposed to the electrolyte, allowing Li^+^ (de)­insertion (Figure 1c). The basal plane (0001), which is impermeable to Li^+^ exchange,^44,45^ represents only 2 out of 8 of the crystal surfaces in the octahedron morphology. Throughout this work, the specified particle sizes refer to the edge length of the octahedron, as depicted in Figure 1c. A significant number of the nominally octahedral particles in the sample are not perfect, with defects, missing parts of the particle, or having epitaxial growth of different oriented particles on the sides, resulting in multiple grain boundaries within the same particle, which become more visible after cycling, particularly if cracking has occurred (Figure S2). Some octahedra appear truncated, likely because the particles have broken, as visible from the additional SEM images (Figure S3). However, for the purposes of our study, we will consider any facet that exposes transversal layers as a side plane, since its behavior is qualitatively similar to that of the (01–12) side plane with exposed Li-containing layers.

### 3.2 Charge Photometry

We then employed operando CP to image the different facets during battery operation and study the facet-dependent Li^+^ diffusion by analyzing the scattered intensity.^12,34,46^ Figure 1d shows raw optical responses for the basal plane (red curve; see the SEM image of the exact particle in Figure S4a) and from the side planes of a particle with multiple grains (blue curve; see the SEM image in Figure S4b), superimposed with the voltage profile. Two typical representatives of basal and side planes are shown in Figure 1e,f. Consistent with previous studies that also examined the optical intensity from basal planes of single-crystal NMC 811 particles, which showed that the lithiation state of the individual particles can be inferred from the optical intensity for NMCs,^12^ the optical intensity increases with decreasing degree of lithiation.

It is also important to consider that the light (740 nm) used in the optical experiments can only penetrate to a certain depth, thus probing the surface and subsurface lithiation state of the particle.^34,39^ Assuming our material has a very similar absorption to stoichiometric NMC811, we estimate that light penetrates approximately 100 nm for the side plane and 50 nm for the basal plane, as shown in ref 39. The penetration depth has a significant impact on the observed behavior of the side planes; however, the trends observed for the basal planes are less dependent on the penetration depth.

Notably, the basal plane demonstrates a significantly higher brightness (intrinsically higher initial optical intensity) throughout the cycle in comparison to the side plane. This difference may be attributed at least in part to the much rougher surface morphology of the side planes in these particles (Figures 1f and S3), which can scatter light outside the collection aperture of the objective, resulting in reduced intensity counts. In order to investigate and compare their behaviors, the subsequent optical response curves are normalized to the final frame with the assumption that all particles achieve equilibrium in the lithiation state at the end of an electrochemical experiment that finishes with a rest/voltage hold.

### 3.3 Charge-Rest (GITT) Experiments

To investigate the mechanisms behind the different optical responses of the two distinct particles’ planes in greater detail, we conducted a series of charge-rest experiments, which qualitatively simulate GITT experiments performed to estimate Li^+^ transport behavior. During these experiments, a current pulse of 1C was applied for 60 s to delithiate particles by a small amount corresponding to composition change of approximately *i* = 0.017 for Li_1–*i*_ TMO_2_, across all the particles in the electrode. This was followed by a 10 min rest period, during which a change in the lithiation state of the particles is not commonly anticipated or assumed in most analyses of GITT data.^37,38^ (The full electrochemical profile is given in Figure S5 with a 3.6 mAh/g charge inserted per pulse.) The 1C current pulse ensures a strong, high signal-to-noise optical response while maintaining relevance to commercial lithium-ion battery charging protocols.^47–49^ Figure 2 shows the evolution of the integrated intensity of either the basal or side planes of differently sized particles under the charge-rest electrochemical protocols. Overall, the intensity of particles larger than 10 μm showed a sharp increase during the first current pulse, corresponding to a decrease in the lithiation state (increase in the SOC, Figure 2a). After the end of the current pulse and during the rest period, the side plane particles exhibited a rapid drop in optical intensity, as seen in Figure 2a. This is consistent with relaxation during the rest period where the particles start to equilibrate and Li^+^ ions diffuse from the center of the (in this case, approximately 11 μm) particle toward the faces and into the depth of view of the optical experiment (approximately 100 nm), resulting in a drop in intensity (increased lithiation). This is consistent with the general interpretation of a GITT experiment: During the current pulse, Li is removed from the sides of the particle, causing a concentration gradient within the particle, which is relaxed during the rest period. Interestingly, and unexpectedly, an increase in optical intensity was observed during the first rest step for the basal plane particles, corresponding to a decrease in the overall Li^+^ content of the Li^+^ subsurface layers being observed in this experiment, even though no external current is being applied (Figure 2a). These two distinct optical responses reflect the same delithiation mechanism but are observed from different perspectives (or fields of view). The two different facets of the particles were identified and confirmed by subsequent SEM imaging (Figure S2a,d, Note 2).

**Figure 2:**
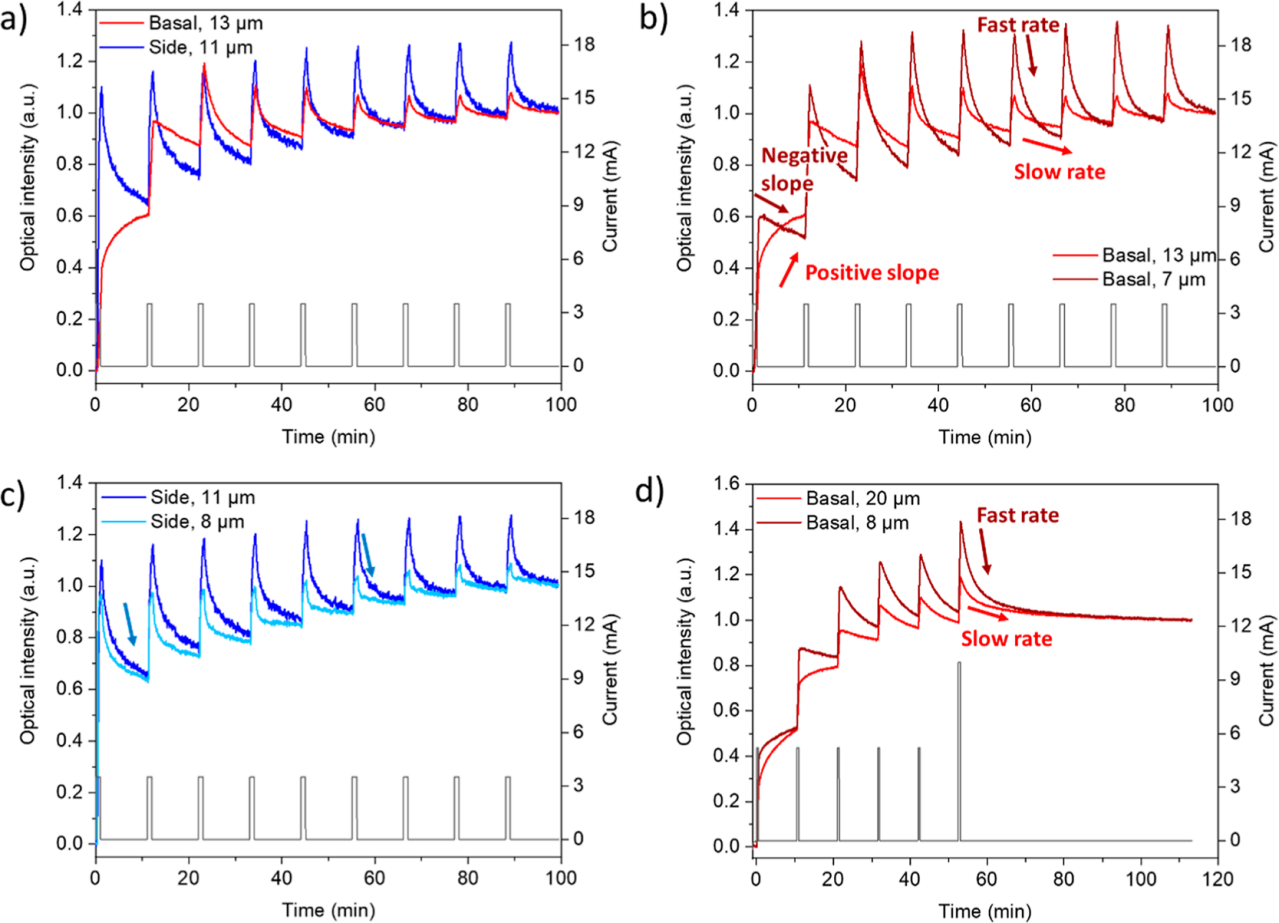
(a) Optical intensity curves (normalized by final frames) representing particles oriented with basal and side planes visible by CP, when pulsed with 1C pulses for 1 min, with 10 min rests in between. The first pulse is set to 0 s at the start of the charge-rest experiment. A C/3 discharge to 3 V, followed by a 30 min potential hold at 3 V, was performed before these experiments commenced in order to lithiate the particles. (b,c) Further optical intensity curves for different particles within the same electrode but with varying sizes and different orientations. (d) Experimental results performed using a 1C pulse for only 30 s followed by a 10 min rest. A final pulse of 2C for 30 s was used; the final rest period is 1 h long. The basal planes of a large and a smaller particle are compared. The black traces in all the graphs represent the current pulses. Arrows highlight differences in relaxation behavior during the rest periods.

Particle size was also found to have a noticeable influence upon the optical behavior, with two different regimes being observed in the case of basal planes. As seen in Figure 2b, for larger (13 μm) particles, the optical intensity continues to increase during the first few rest steps, which we ascribe to a continuation of the delithiation even during rest. This is followed by a regime where relithiation of the particle surface (decrease in the optical signal) was observed during the remaining rests (see bright red arrows). In contrast, smaller particles with a limited Li^+^ reservoir exhibited a more rapid increase in intensity (delithiation) during the charging pulse, the relithiation behavior starting from the first rest (see dark red arrows). By contrast, the side planes of differently sized particles do not differ as significantly in their optical behavior (Figure 2c, S2b,d), as both sizes increase in their intensity sharply during the current pulse (due to the delithiation of the particle facets). This is followed by rapid drops during the rest periods (see blue arrows), as Li^+^ ions move from the bulk to the subsurface region, probed optically, to replenish the vacated Li sites. These results highlight the role of particle size in governing the electrochemical and optical behavior with respect to the rest of the particles in the entire electrode. A wider range of particles was explored (Figure 2d, showing an experiment that included a different charging protocol and a longer resting step at the end, as well as the Supporting Information, such as Figure S10 and Figure S11), and even though some of the particles show flaws, including cracks and multiple grain boundaries (Figure S3), their behavior is well described with the general trends described here.

### 3.4 Simulations of Charge Photometry Behavior

Simulations were then used to understand the evolution of the scattered intensity from the different planes of these particles. A perfect octahedron was assumed to simplify the model. While the side planes of the modeled particle correspond to the {01–12} facets, they can be used as a proxy for any facet that dissects the crystallographic (0001) layers. First, we considered a hypothetical scenario where no (de)­intercalation of Li^+^ ions through the particle’s surfaces takes place during rest steps, with ion transport restricted to diffusion within the layers of the particle.^26^ In this approximation, we assumed that the current density across all of the side planes (active surface) of the octahedron is uniform (and hence equal to zero everywhere during the rests). Figure 3a,b shows the evolution of the lithiation state across the volume of the particle and on the surfaces when this condition is enforced. In this case, starting from a uniform lithiation state, a rapid delithiation of the near-surface region of the side planes is observed during the first pulse (Figure 3a, 60 s). This translates into a rapid and large change of the average θ-value (Li^+^ concentration) at the side surfaces, as compared to the much smaller change on the basal planes, which (when integrated across the whole plane) corresponds exactly to the total Li extracted from that layer during the pulse (Figure 3b, 60 s and Figure 3c, first current pulse). This difference originates from the fact that all of the side plane surfaces can be delithiated while only the edges of the basal plane can.

**Figure 3:**
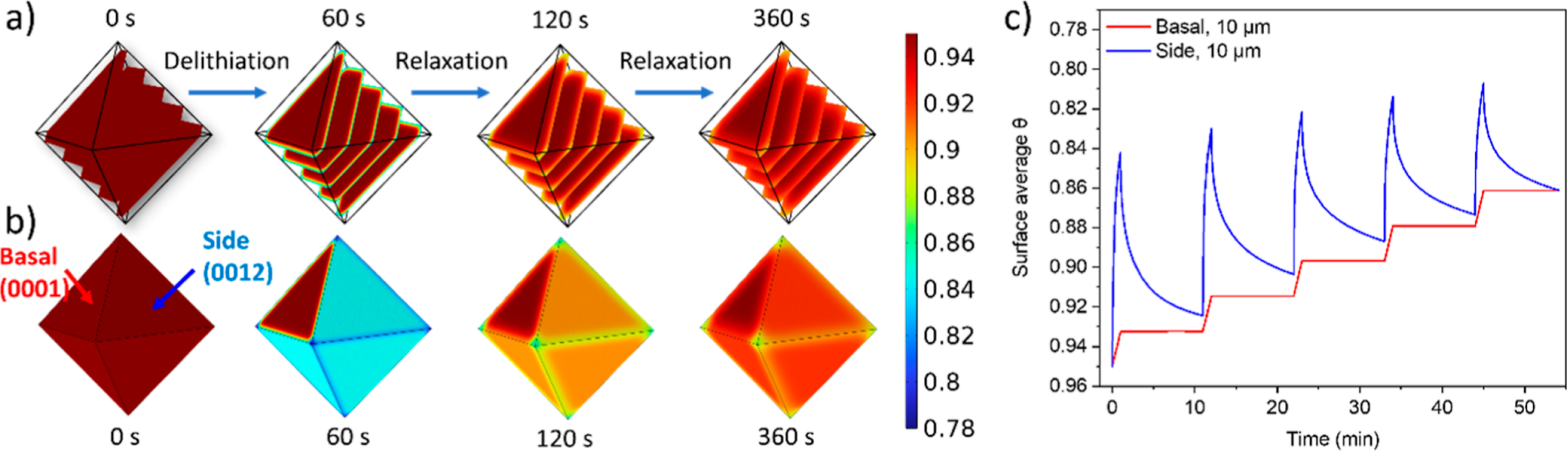
Simulation results for pulse-rest protocols of one octahedral particle enforcing a uniform current density across the active surfaces (side planes), for a pulse-rest protocol consisting of 1C pulses for 1 min, followed by 10 min rest. (a) Representation of θ values at different times before (0 s) and after (60 s) and during the first rest period (120 and 360 s), showing the variations of θ across the *ab* (0001) Li layers and (b) on the exposed surfaces. The same scale bar (shown on the right-hand side of (a,b)) is used for both these figures. (c) θ values averaged over exposed basal and side planes (i.e., the surface average θ). The lithiation state axis is represented from higher to lower values to allow for easier comparison with the experimental intensity evolution. Simulations start from a composition of θ = 0.95, since in practice it is difficult to fully relithiate an NMC811 particle.^40^

During rest, a redistribution of the Li^+^ across each of the layers of the crystal takes place (Figure 3a, 60 to 360 s). This redistribution within the layers translates into a change of the lithiation state of the side planes during relaxation, as Li^+^ diffuses to the surface from within the crystal (Figure 3b, 60 to 360 s and Figure 3c, first rest). Since there is no (de)­intercalation of Li^+^, the total amount of Li^+^ within each layer remains constant and hence the average *q* on the exposed basal plane surface does not change (Figure 3c, first rest). Note that equilibrium is still not reached after the rest period, largely due to the slow Li^+^ kinetics at this SOC. This response is repeated on each consecutive pulse-rest, where the same effects can be observed.

The behavior observed when no (de)­intercalation is allowed during the rest period captures the main features experimentally observed for the intensity changes on the side planes (comparison Figures 2c and 3c), which implies that the behavior of θ on these surfaces is dominated by the solid-state Li^+^ diffusion processes from the bulk to the surface. However, the fact that the experimentally inferred lithiation state of the basal plane changes during the rest steps, as evidenced by the change in intensities seen in the CP experiments (Figure 2b), is inconsistent with the predicted behavior if no (de)­intercalation of Li^+^ happens during rest.

Hence, this suggests that Li^+^ ions undergo intercalation and deintercalation either between different layers within the same particle, between different grains within the multigrain particles, or between different octahedral particles during the relaxation steps.

The second approximation used to model the octahedral NMC behavior allows for the (de)­intercalation of Li^+^ within the same particle but not between different particles (i.e., Li^+^ can exchange between central and outside layers, i.e., layers closer to the basal plane facet). To achieve this, the voltage within the electrolyte is taken to be spatially uniform, and a single particle is simulated where the current density across the surface is left free to take different values that integrate to the value of the overall expected current (eq 7). This is illustrated in Figure 4a and Figure 4b, which show two models of a 5 μm particle, displaying the variation of θ values and current values on the surface of the particle at two different time stamps (120 and 360 s) during a first rest period. These evolutions of the average θ values for the basal plane particles with edges that are 5 and 10 μm in length in such a scenario are also shown in the form of evolutions over time in Figure 4c. Here, a small change in the lithiation state of the basal planes can be observed during rest, given by “intraparticle” cross-talk. It is interesting to note that this cross-talk effect shows a small size-dependency. This effect originates from the geometry of the particles: the central Li layers are hexagonal in shape, while the external layers are triangular, both sharing the same perimeter length but different areas (thus different total Li contents). Hence, the central layers have a higher area-to-perimeter ratio than the outer layers. This leads to inhomogeneous lithiation rates across the particle during the pulse steps, which equilibrates during the relaxation steps via cross-talk between layers, leading to a nonconstant average θ on the basal planes. This approximation, however, does not capture the significantly different cross-talk rates for differently sized particles following pulses 6 to 10, where the rates of decay in the optical images of the basal planes depend strongly on size (Figure 2b, “slow rate” and “fast rate” arrows), while in simulations the decay is slow with little size dependence (Figure 4c, “slow rate” arrows).

**Figure 4:**
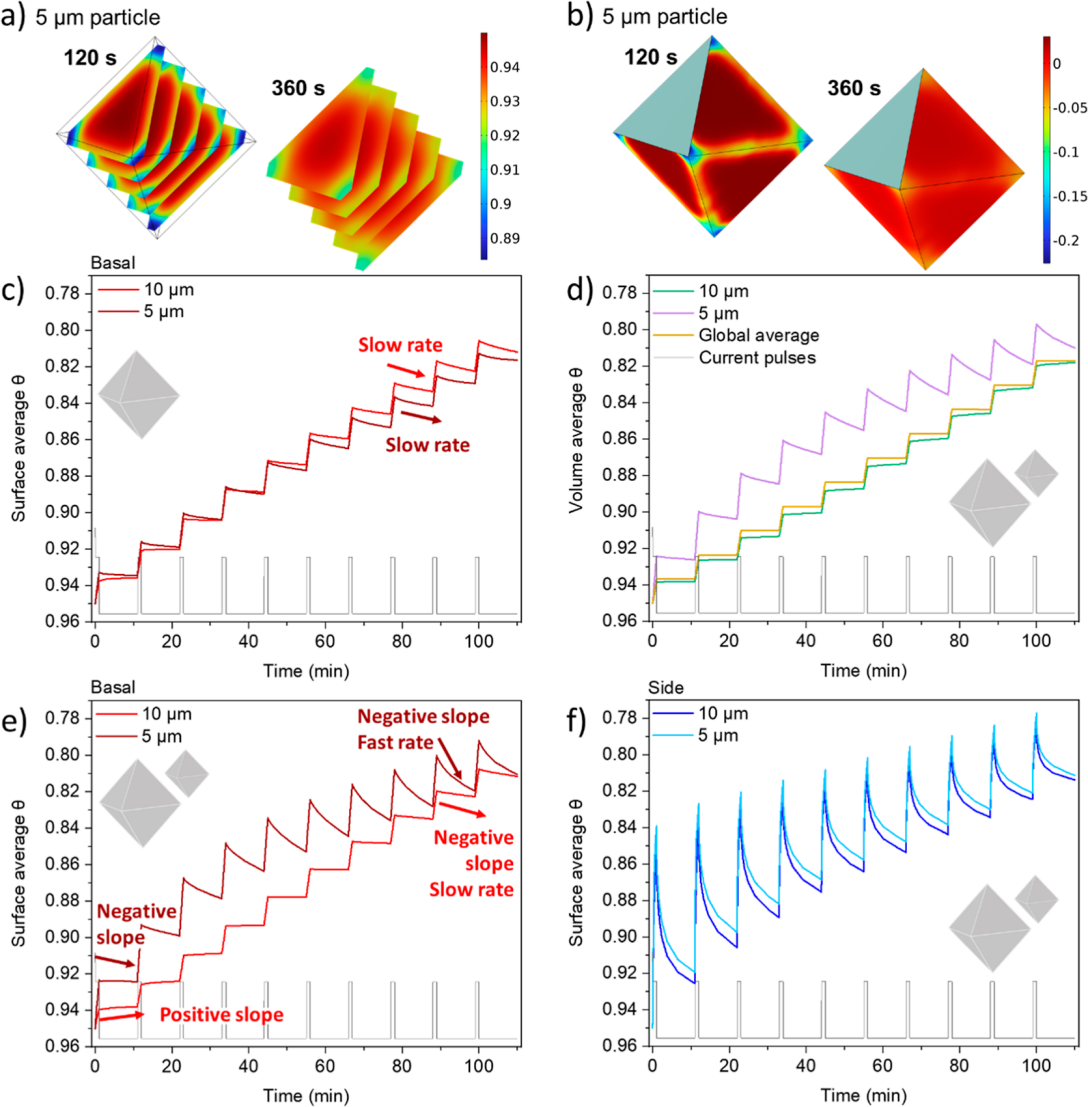
(a–c) Results for pulse-rest protocols of single-particle simulations, allowing for (de)­intercalation of Li^+^ within the same particle (“intraparticle cross-talk”). (a) Representation of θ values at two different time stamps during the first rest period (120 and 360 s), showing the variations of θ across the ab layers. (b) Representation of current values at two different time stamps during the first rest period (120 and 360 s), showing the variations of current on the exposed surfaces. (c) Surface-averaged θ evolution on the basal plane face for the cases of two differently sized particles. (d–f) Results for two-particle system simulations, allowing for “interparticle cross-talk”: (d) the volume-averaged θ evolution of each particle along with the global one (i.e., the average over both particles), (e) the surface-averaged θ evolution on the basal plane face for each particle in the system, and (f) the side plane surface average of θ for each particle. All lithiation state axes are represented from higher to lower values to allow for an easier comparison with the experimental intensity evolution. The black traces in all the graphs represent current pulses during the simulations. The pulse protocol consisted of 1C pulses for 1 min, followed by 10 min rest in all cases.

### 3.5 Interparticle Communication

The scenario where only intraparticle cross-talk is considered is only appropriate in the case of single-particle electrodes. Furthermore, if Li^+^ is able to exchange between layers of the same particle, it is reasonable to expect that it also transfers between different particles via the liquid electrolyte, as long as the particles are electrically connected. The third approximation, then, is to account for interparticle cross-talk. Finite element simulations of a two-particle ensemble of one 5 and one 10 μm particle were then performed, where we allow for (de)­intercalation of Li^+^ from one surface site/particle to another. The volume-averaged value of θ for each particle obtained in these simulations (Figure 4d) shows clearly that the small (5 μm edge) particle is more delithiated during each pulse, consistent with the higher intensity changes observed in the experiments for the basal planes of the smaller versus larger particles (Figure 2b,d). This is reasonable since the smaller particle has a higher surface-to-volume (S/V) ratio, and hence, in relative terms, delithiation occurs faster. During the rest of the steps, both particles equilibrate by a transfer of Li^+^ ions from one particle (10 μm) to the other (5 μm). The effect of exchange on the average θ in the smaller particle is much more pronounced because the same amount of transferred ions contributes to a much larger portion of its Li^+^ reservoir compared to the larger particle.

Figure 4e,f shows the evolution of the average lithiation state seen on the different surfaces obtained in the two-particle ensemble simulations. By comparing a basal plane with a side plane, a trend similar to experiments can be observed (Figure 2a). The side planes experience much more drastic changes in the lithiation state, but they are still dominated by intraparticle solid diffusion (Figure 4e). The changes seen between particles on the basal planes, although smaller, are significant. The main features of the experimental intensity profile (Figure 2b) are well reproduced in Figure 4e: bigger particles show two distinct regimes originated by interparticle communication; a first regime where the basal plane continues to delithiate during rest (“positive slope”, bright red arrow) and a second one where it relithiates (“negative slope”, bright red arrow); by contrast, smaller particles only evidence the second regime (“negative slope”, dark red arrow). Furthermore, the cross-talk rate during the sixth to tenth rest periods is significantly faster for the 5 μm particle as compared to the 10 μm one (“fast rate” and “slow rest” arrows), confirming that interparticle cross-talk needs to be accounted for to explain the experimental results.

To explore the origin of the two regimes shown in Figures 2b and 4e further, exemplified by the average θ evolution for the larger particle’s basal planes, the spatial distribution of current density in the two-particle simulation was examined, as shown in Figure 5. This is shown at different times during the pulse-rest protocol. During the first pulse, at 50 s, the current density is higher overall in the smaller (5 μm) particle, where the S/V effect is largely seen as an edge effect (Figure 5a,e). During the eighth pulse, this is reversed, and the larger (10 μm) particle experiences a higher overall current density (Figure 5c,f). These effects originate from a competition between bulk diffusion and surface (de)­lithiation kinetics. It is known that the diffusion coefficient of Li^+^ in the NMC lattice drops when approaching the fully lithiated state, and a similar effect happens to the exchange current density, *j*_0._ ^26,50^ A physically meaningful comparison of *D* and *j*_0_ can be achieved by calculating the equivalent length, *L* = *D*·*F*·*N*_tot_/*j*_0_. This length can be interpreted as the extra distance ions would need to travel in a particle with no intercalation resistance to experience the same overall resistance, in essence, translating kinetic limitations into an equivalent diffusion length.

**Figure 5:**
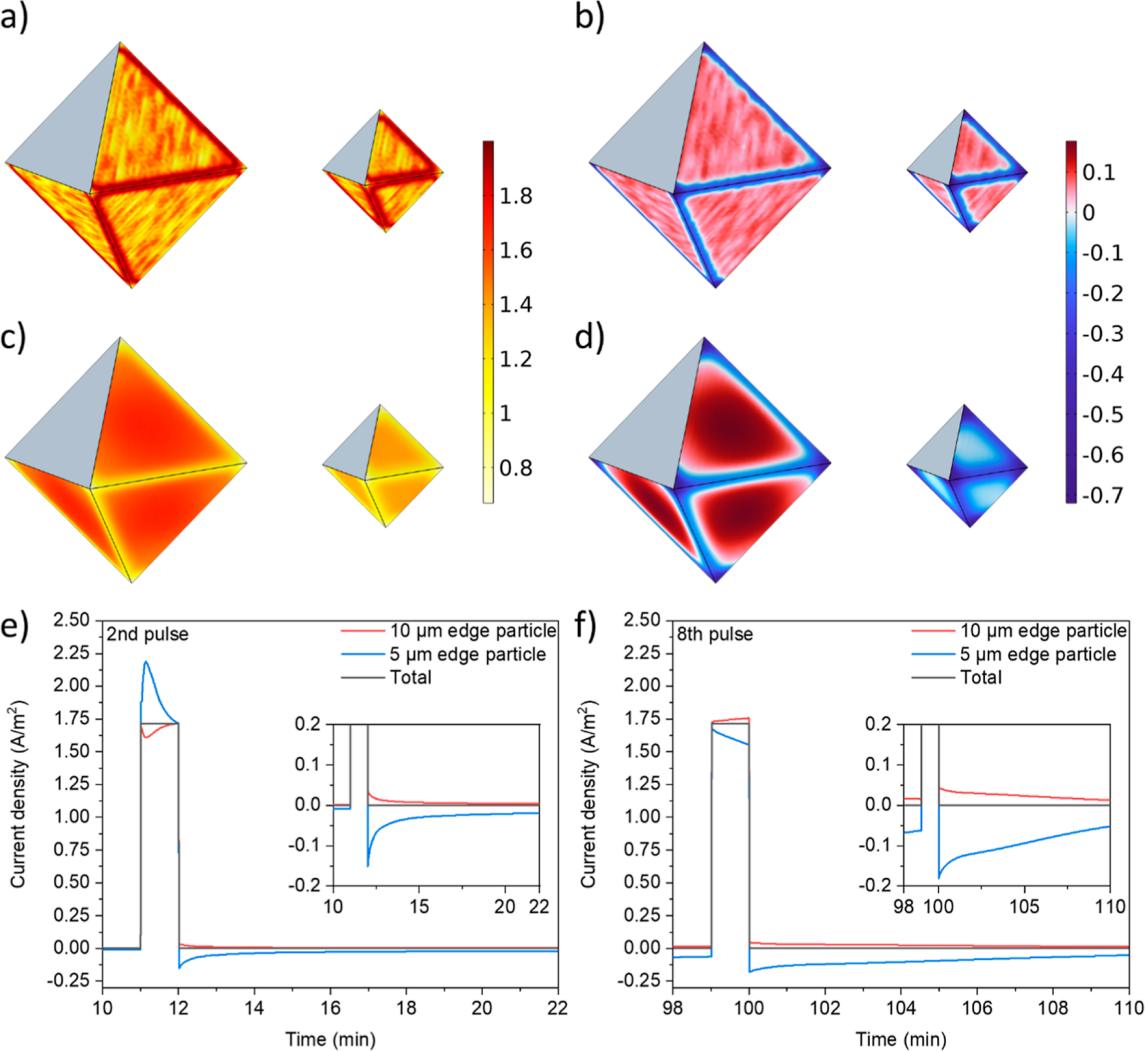
Results for idealized models showing the local current density at the surfaces of two electrically connected, differently sized particles (10 and 5 μm edge sizes), at different time points during the charge-rest procedure: (a) first and (c) 8th pulse after 50 s and (b) first and (d) 8th rest period after 10 s. The (positive) color scales for both (a,c) are shown on the right-hand side with values shown in A m^–2^; panels (b,d) share the same (now positive and negative) color scale, shown to the right of these panels. The inactive basal planes are shaded gray. Average current density across the side planes of each of the particles during (e) the second and (f) the eighth pulse-rest periods. Insets represent a zoom of the current–time plot during rest.

Comparing *L* to the actual particle size provides insight into whether the intercalation kinetics or solid-state diffusion is the dominant limiting factor in lithium transport. *L* is plotted in Figure S6c, and it is notable how *L* shows a sharp decrease from the near-full lithiation state to θ = 0.8 (from 10 μm, similar to the experimental particle mean size, to 1 μm, much smaller), implying that the system shifts from a surface kinetics-limiting regime to a bulk diffusion-limiting regime. This is a consequence of the rapid increase of *j*_0_ upon delithiation,^26,43^ the rate of change in *j*_0_ being larger than that of the diffusion coefficient^40^ (Figure S6). Results for simulations of pulse-rest protocols, including kinetic and/or diffusion limitations, are analyzed in Note 3 and Figure S7.

It has been reported that the kinetically limited regime leads to an “autocatalytic” behavior, where particles that delithiate first, then have accelerated kinetics over those with higher states of lithiation.^26^ The same effect leads to inhomogeneities in the lithiation state across the surface, as observed in Figure 5a, where the edges of the particles show enhanced delithiation rates during the first pulse, and in Figure 5e where it is evident that the average current density in the smaller particle is higher during the first few pulses. After the overall surface lithiation state reaches around 0.88 (also see Figure S8), the kinetic limitation is relaxed, which is supported by the decrease of the equivalent distance *L* (representing a measure of the intercalation resistance), from 120% to 13% of the average particle size between θ = 0.95 and θ = 0.86 (Figure S6c). Now, the diffusion-limited regime starts to be apparent. The current density across the surface becomes more homogeneous, as shown in Figure 5c (8th pulse), and the average current density during the pulse in the small particle is now lower (Figure 5f).

During the rest periods, the “interparticle” exchange can be observed in the negative and positive average current densities and total current for the 5 and 10 μm particles (Figures 5e,f and S9). “Intraparticle” communication is observed in the current density distribution during the rests (Figure 5b,d), where the points and edges of the octahedra experience negative current (relithiation), whereas the middle of the faces shows positive current (delithiation).

The extent of intra- and interparticle cross-talk appears to depend on the amount of charge passed during the pulse (i.e., total Li extracted) but not on the pulse rate, as shown in simulations conducted at different rates (Figure S10a,b). Experiments performed at different rates and pulse lengths confirm this hypothesis, evident mainly in Figure S10c,d. To determine whether heterogeneity between particles could be related to electrolyte transport limitations, where particles closer to the separator are delithiated before the ones that are farther away, the optical experiments were repeated on a thinner electrode (40 μm compared to a normal thickness of 150–200 μm). However, for the experimentally accessible particles (i.e., those seen optically), the effect is negligible, as the same trends were observed (Figure S11). A purely geometric explanation is therefore much more likely to be the origin of this phenomenon, at least in these electrode formulations.

### 3.6 Implications of the Model

As established in the previous section, the expected extent of interparticle communication is directly related to the lithiation heterogeneity developed during the (de)­lithiation process, which is influenced primarily by particle sizes. Hence, in this section, we explore the sensitivity of interparticle cross-talk to the standard deviation and mean of different particle size distributions.

Systems composed of 10 octahedral particles were simulated. The particle sizes of each 10-particle ensemble were generated to represent different log–normal distributions, as seen in Figure 6a. Simulations for each of these systems were performed following the same charge-rest protocol as the previous section 3.5: 1 min current pulses at 1C, followed by 10 min rests at total current = 0, assuming Li^+^ transport in the electrolyte is not limiting.

**Figure 6:**
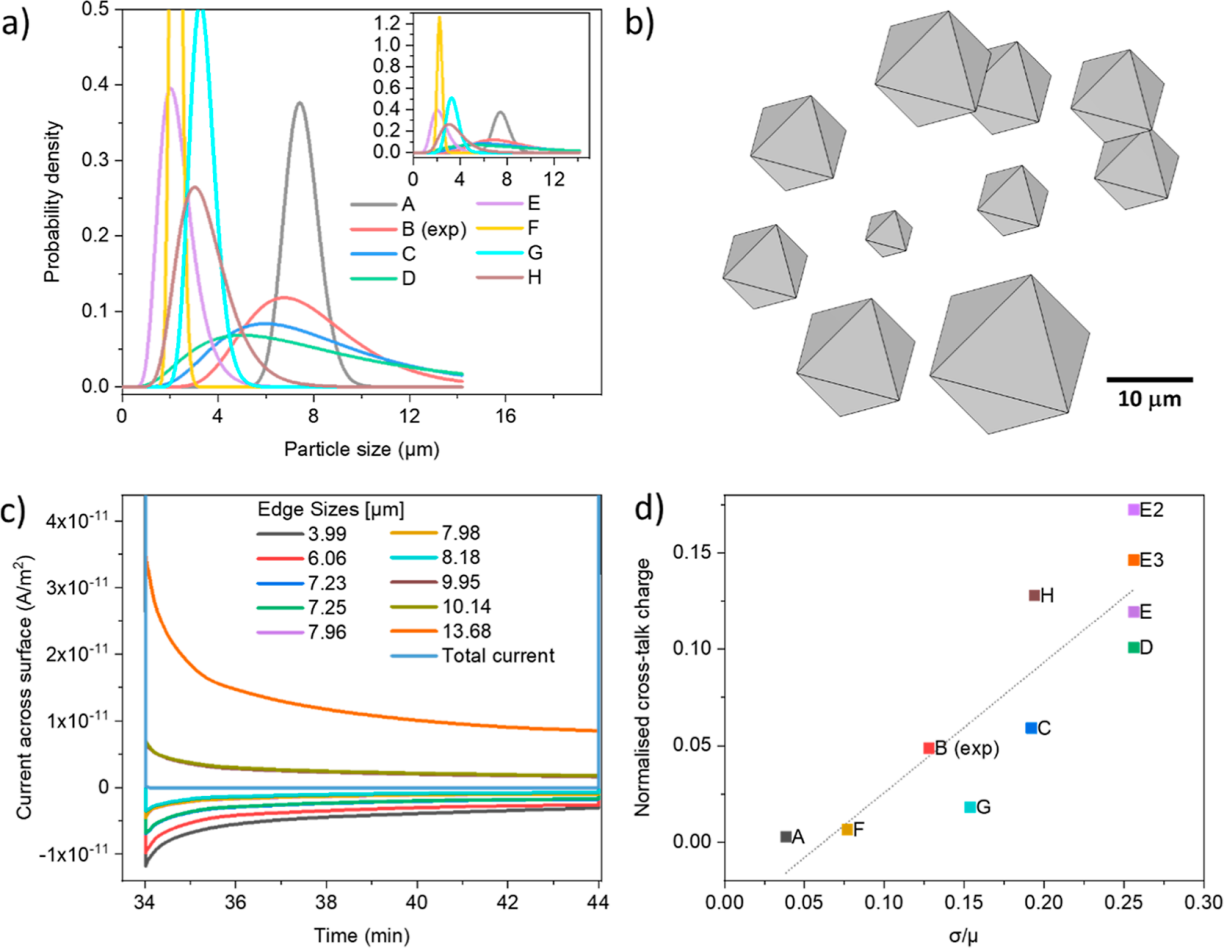
(a) Particle size distributions (PSDs) chosen to generate the 8 sets of 10 particles with different distributions of edge lengths used in the simulations. The parameters to generate these distributions can be found in Table S1. B (exp) represents the experimental particle size distribution. (b) Example of particle ensembles composed of 10 particles representing the experimentally obtained particle size distribution, B (exp). (c) Current across each of the particles’ active surface during the fourth rest for distribution, B (exp). (d) Cross-talk charge (integrated current during rest) normalized by pulse capacity after the fourth simulated pulse as a function of the relative dispersion of the particle population, σ/μ. Each data point in (d) represents one of the distributions in (a); the three data points E, E2, and E3 correspond to simulations from three different sets of particles that were generated for E.

The current across the surface of each particle during the pulses and the rest was obtained from each of these simulations (Figure 6c), as well as the average θ values across the basal plane of five selected particles (Figure S12). The cross-talk current during rest is defined as half the sum of the absolute values of the current in each particle. By integrating these values during the rest period, an effective “cross-talk capacity” is extracted, which is related to the number of Li^+^ ions that is deintercalated from one particle and intercalated into another.

Figure 6d shows the cross-talk capacity, normalized by the pulse capacity, as a function of the relative dispersion of the particle population. This dispersion is represented using parameters of a log–normal distribution: the shape parameter, σ (the standard deviation of the underlying normal distribution) and the location parameter, μ (the mean of the underlying normal distribution). A higher σ/μ ratio indicates a greater relative dispersion within the population. The analysis clearly demonstrates that increasing size dispersion significantly enhances cross-talk capacity—reaching up to 15% of the pulse capacity under the simulated conditions. The cross-talk capacity depends heavily on used experimental conditions, especially the relaxation time during rest periods. If the relaxation period is too short, it will increase the cross-talk in later pulses (see Figure S13) and further limit the accuracy of the apparent diffusivity extracted from the GITT method. The cross-talk effect is governed by diffusion dynamics and reaction kinetics and is expected to vary with SOC. Also, it is likely to be most pronounced at low SOC, near full lithiation, where kinetic limitations and spatial gradients are strongest. Investigating how cross-talk evolves over the full charge–discharge cycle and how it is influenced by different testing protocols is an interesting pathway for future work.

We highlight that to describe and compare these effects across systems properly, it is essential to characterize particle size dispersion using parameters appropriate to the actual population distribution, in this case, log–normal. Using inappropriate statistical descriptors—for example, calculating a standard deviation under the assumption of normality (Gaussian)—can lead to misleading conclusions and poor predictive reliability.

The charge–rest experiments performed and simulated in this work bear resemblance to GITT protocols. Our findings indicate that the relaxation behavior observed during rest steps commonly used to extract the diffusion coefficient is influenced not only by solid-state lithium diffusion within the single-crystal particles but also by lithium exchange processes at the particle–electrolyte interface (either between different particles or between different grains in particles with more than one grain). As a result, diffusion coefficients derived from the GITT data should be interpreted as apparent values that incorporate both bulk diffusion and interfacial exchange effects. The contribution of Li^+^ exchange is expected to be more pronounced in samples with more broadly distributed particle populations, where interparticle variability enhances the contribution of exchange phenomena during relaxation.

Finally, the observed SOC heterogeneity within the particles may also lead to additional sources of mechanical stress during cycling and different modes of degradation. For example, in charge–discharge protocols involving short pulses, commonly used in real application scenarios, the smaller particles will experience larger, more rapid variations in SOC, triggering degradation processes, such as oxygen loss and coupled electrolyte degradation. Furthermore, strategies for electrode engineering involving blending small and large particles, single crystals, and polycrystalline particles or even different chemistries will show strong particle cross-talk effects, and their impact on the performance and degradation remains to be understood. In these cases, considering that the larger particles are more likely to undergo lithiation-gradient-induced cracking (particularly at higher rates), if the smaller particles represent a significant volume fraction, it is possible that, since they (de)­lithiate more rapidly, they may prevent sharp gradients from occurring in the large particles, thereby acting as buffers, potentially reducing cracking.

## 4 Conclusions

In this study, we employed charge photometry (CP) and finite element simulations to investigate the behavior of two distinct crystallographic planes in octahedrally shaped cathode active materials (with composition close to NMC811), focusing on lithium-ion diffusion during charge-rest protocols. By using active electrode material particles with well-defined structures, we could systematically study various phenomena related to the different distinctive crystallographic facets. This enables the investigation of both single- and multiparticle behaviors using a combination of characterization techniques, electrochemical measurements, and modeling approaches. Our findings reveal differences in optical behavior between the planes, underscoring the anisotropic nature of Li^+^ dynamics within the crystals.

By combining charge-rest experiments with theoretical simulations, we examined and rationalized the delithiation behavior of the NMC particles. We found that particle size distributions, together with kinetic and diffusion limitations, significantly influence the lithiation dynamics. The application of small current pulses leads to significant differences in the extent of delithiation, with more lithium being removed from smaller particles due to their larger surface to volume ratios. Most notably, during rest steps in the charge-rest experiments, Li^+^ ions equilibrated both within the particles and between particles through the electrolyte. This interparticle communication was shown to play a crucial role in controlling Li^+^ dynamics at the single-particle level. Practically, this effect will complicate the analysis of methods such as GITT, a standard method often used in the battery field to extract diffusion coefficients. The observed state of charge heterogeneity within particles may also affect the sources of mechanical stress during cycling and lead to different modes of degradation.

## Supplementary Material



## Notes

Simulation procedures and detailed notes, PSD plot, SEM images, simulations of different particle systems, current plots, and optical experiments (PDF)
