# Supplementary material for: Interparticle Communication and Lithium Dynamics in Faceted Nickel-Rich NMC Cathodes
Source: J Am Chem Soc. 2026 Jan 21;148(4):4097–109. doi: 10.1021/jacs.5c15171 (PMC12879733; doi:10.1021/jacs.5c15171)
Supplement: Supplementary file 1 [file ja5c15171_si_001.pdf]

# Inter-particle communication and lithium dynamics in faceted nickel-rich NMC cathodes

Veronika Šedajová,[1,#] Gabriela Horwitz,[1,2,#] Jiho Han,[1,3] Alice J. Merryweather,[1,3,5] George S. Phillips,[1,2] Vikram S. Deshpande,[2,4] Norman A. Fleck,[2,4] Akshay Rao,[2,3] Clare P. Grey,[1,2,*]

1. Yusuf Hamied Department of Chemistry, University of Cambridge, Lensfield Road, CB2 1EW, Cambridge, UK

2. The Faraday Institution, Quad One, Harwell Science and Innovation Campus, OX11 0RA, Didcot, UK

3. Cavendish Laboratory, University of Cambridge, J.J. Thomson Avenue, CB3 0US, Cambridge, UK

4. Department of Engineering, University of Cambridge, Trumpington St., Cambridge, CB2 1PZ, UK

5. Illumion Ltd., Maxwell Centre, J.J. Thomson Ave, Cambridge, CB3 0HE, UK

*Corresponding author, email: cpg27@cam.ac.uk

#Contributed equally

## Supplementary information:

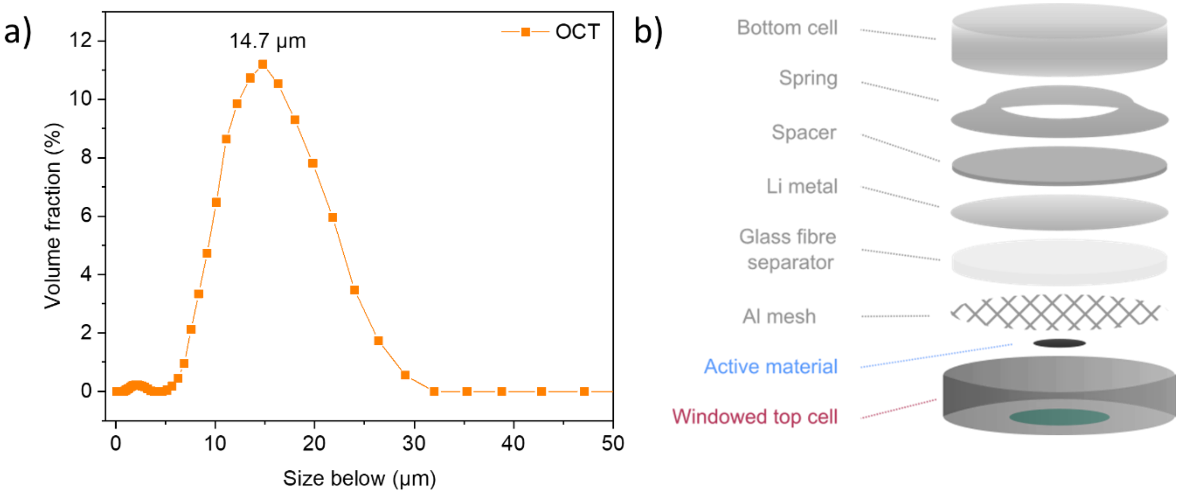


Figure S1: (a) Particle size distribution of the OCT sample. (b) Scheme of an optically accessible coin cell structure.

## Note 1. Simulation methodology derivation:

The methodology used in the simulations and the derivations of relevant equations are briefly summarised, building on the work of Pandurangi et al.[1,2] First, we introduce some notation. We write a superscript A to denote the Li anode, superscript C to denote the NMC cathode particles and superscript E to denote the electrolyte. Write $N_{Li}(x,t)$ as the molar density

(i.e. moles of Li in NMC volume unit) at a given position and time, while $N_{tot}$ is the total number of lattice sites available for Li occupancy, derived from X-ray diffraction (XRD) data and equivalent to 49200 moles $m^{-3}$.[2] Then, define the lithiation state, or lithium occupancy fraction, $\theta(x,t)$, as

$$\theta(x,t) = \frac{N_{Li^+}(x,t)}{N_{tot}} \tag{S1}$$

Throughout the study, the NMC cathode particles are treated as a non-ideal mixture of NMC and lithium and the driving force for diffusion depends on the occupancy-dependent chemical potential of $Li^+$ in the NMC lattice.

At *equilibrium*, all cathode particles share the same state of charge $\theta$, where we use of an overbar to denote the equilibrium state. Then, the equilibrium potential of the cathode is written as $\bar{\phi}^C(\theta)$, the equilibrium potential of the anode is written as $\bar{\phi}^A$ and the equilibrium potential of the electrolyte is written as $\bar{\phi}^E$. The *cell* exhibits an open circuit voltage $V_{OC}(\theta) = \bar{\phi}^C(\theta) - \bar{\phi}^A$. In contrast, $\bar{\phi}^E - \bar{\phi}^A$ is independent of charge.

Now consider the rate of diffusion within a given cathode particle in the non-equilibrium state. The chemical potential of Li ions in the cathode $\mu^C_{Li^+}$ at an electrical potential $\phi^C$ is

$$\mu^C_{Li^+} = \left(\phi^C - \phi^A - V_{OC}(\theta)\right)F + \mu^A_{Li^+} \tag{S2}$$

where $F$ is the Faraday constant, $\mu^A_{Li^+}$ is the chemical potential of Li ions in the anode and $\phi^A$ is the electric potential in the anode. Note that at equilibrium, the above relation reduces to $\phi^C - \phi^A = V_{OC}(\theta)$ and $\mu^C_{Li^+} = \mu^A_{Li^+}$; also note that the chemical potential of Li metal in the anode is $\mu^A_{Li} = \mu^A_{Li^+} - \phi^A F$, leading to the formulation of equation (2) in the main text.

The $Li^+$ flux within the solid cathode, ***J***, is driven by the spatial gradient in chemical potential $\nabla\mu^C_{Li^+}$, again described in equation (2) in the main text.

The flux at the surface of the cathode particles is defined by the Butler-Volmer equation (8), which depends on the lithiation state and the overpotential, $\eta$.

The overpotential $\eta$ is given by

$$\eta = \phi^C - \phi^E - \left(\bar{\phi}^C - \bar{\phi}^E\right) = \phi^C - \phi^E - \left(\bar{\phi}^E - \bar{\phi}^A\right) - \left(\bar{\phi}^C - \bar{\phi}^A\right) \tag{S3}$$

Recall that $V_{OC}(\theta) = \bar{\phi}^C - \bar{\phi}^A$ and $\left(\bar{\phi}^E - \bar{\phi}^A\right)$ is independent of the degree of lithiation $\theta$ of the cathode. Consequently, we can write

$$\eta = \phi - V_{OC}\left(\theta\right) \tag{S4, 9}$$

Where

$$\phi \equiv \phi^C - \phi^E - \left(\bar{\phi}^E - \bar{\phi}^A\right) \tag{S5}$$

We emphasise that $\phi^C$ and hence the voltage difference $\phi$ is spatially uniform throughout the cathode particles but $V_{OC}\left(\theta\right)$ and consequently the overpotential $\eta = \phi - V_{OC}\left(\theta\right)$ varies spatially over the surface of each cathode particle. Consequently, the flux $\boldsymbol{J}$ also varies over the surface of the particle in accordance with the Butler-Volmer relation (8).

**Note 2. Crystallographic orientation of particles**

The crystallographic orientations of the particles investigated by charge photometry were examined by SEM after cycling. Figure S2a shows a basal plane with no clear layered structure visible on the dominant facet oriented upwards. By contrast, a truncated/clipped part (which is clearly not flat or planar) of the particle is visible in Figure S2b. The truncated part likely corresponds to the (110) surface which is also electrochemically active. The basal vs a side could also be inferred in the optical experiments from the absolute intensity values, as the basal plane has an intrinsically higher intensity compared to the side plane, since they are consistently less rough.

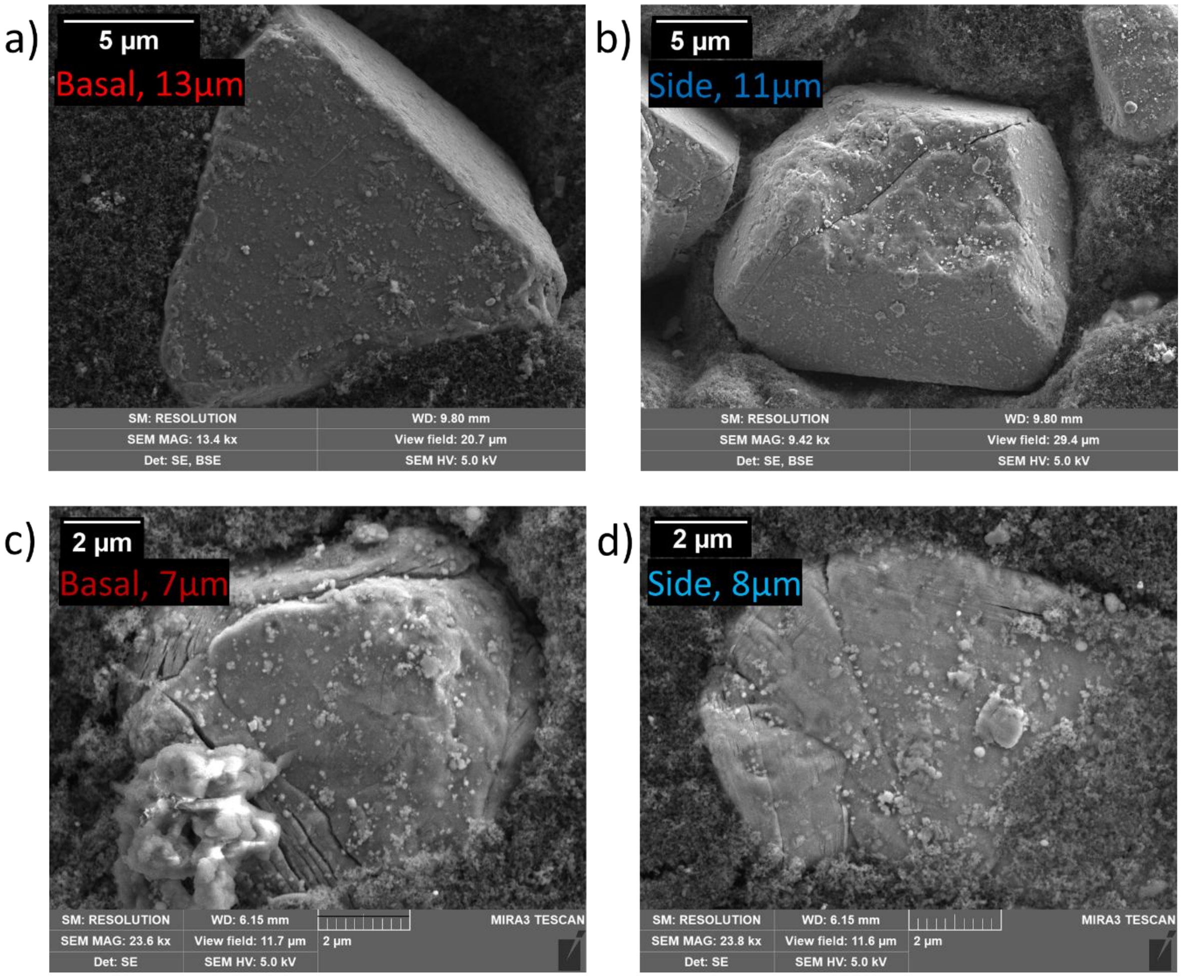


Figure S2. SEMs of the imaged particles after cycling, (a) bigger basal plane (13 µm), (b) bigger side plane (11 µm), (c) small basal plane (7 µm) (only the basal plane part was taken into account for optical intensity extraction), (d) smaller side plane (8 µm).

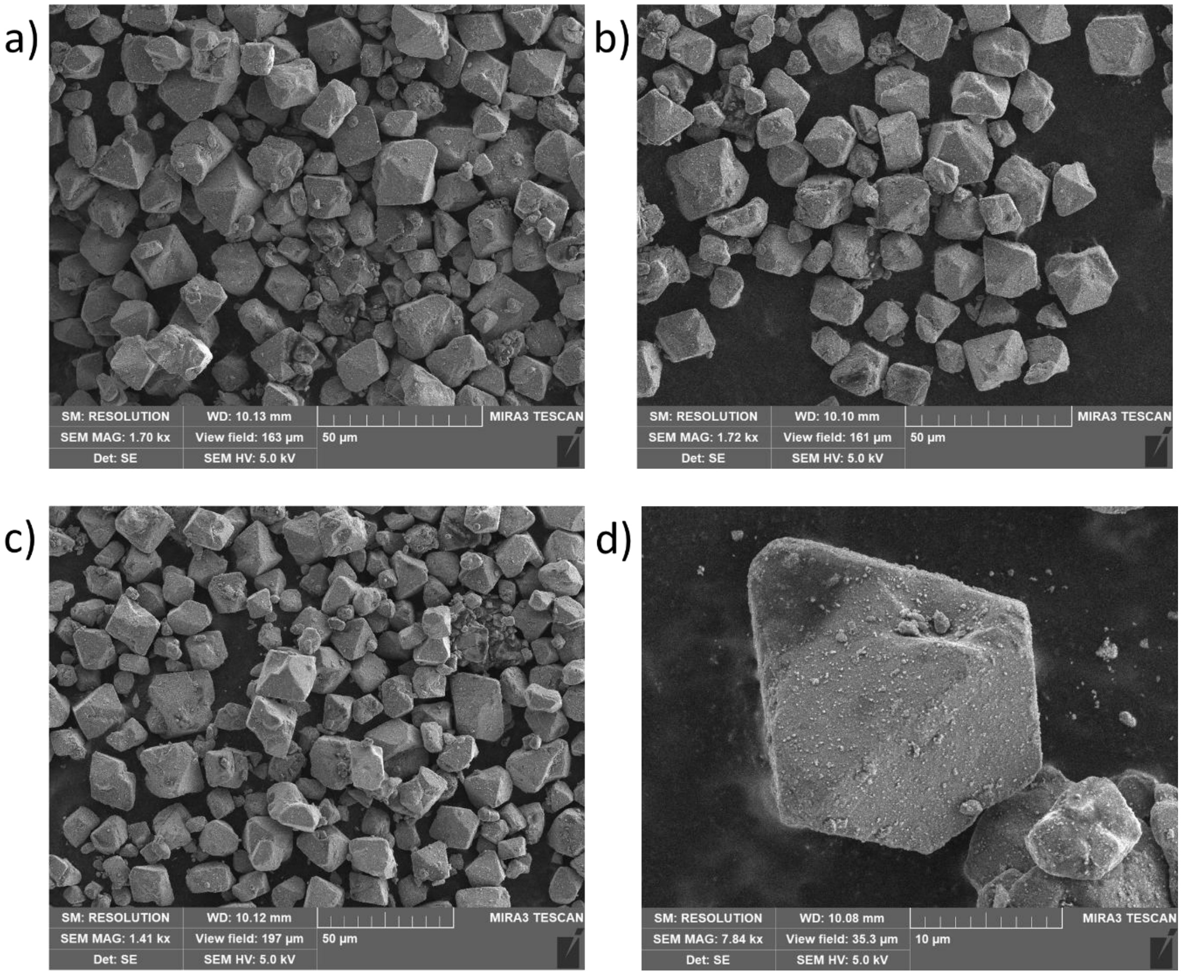


Figure S3. (a–d) SEMs of pristine particles, showing their non-ideal morphology.

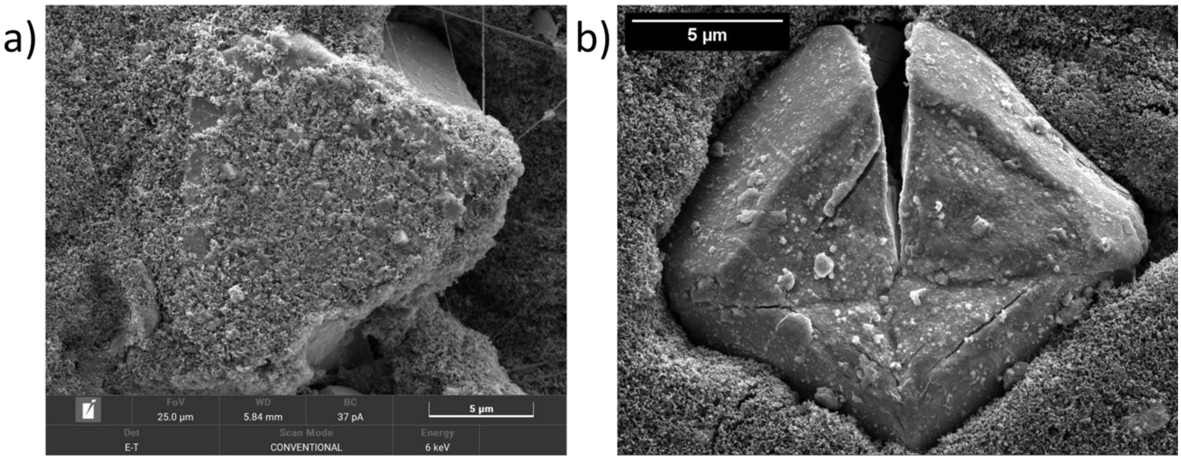


Figure S4. SEMs of the imaged particles after cycling at C/30 rate, (a) basal plane, (b) side plane. The basal plane particle was probably covered in carbon during decrimping of the optical coin-cell.

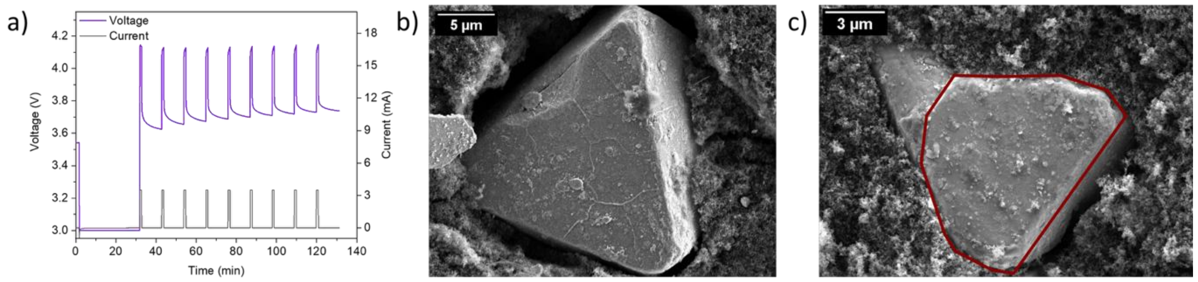


Figure S5: (a) Full voltage and current profile corresponding to 1C for 1 min pulse, 10 mins rest. (b-c) SEM image showing both the 20 µm and the 8 µm particle from Figure 2d. The small particle was only evaluated optically in the region shown, as the upper left part was not within the focus depth of the optical microscope.

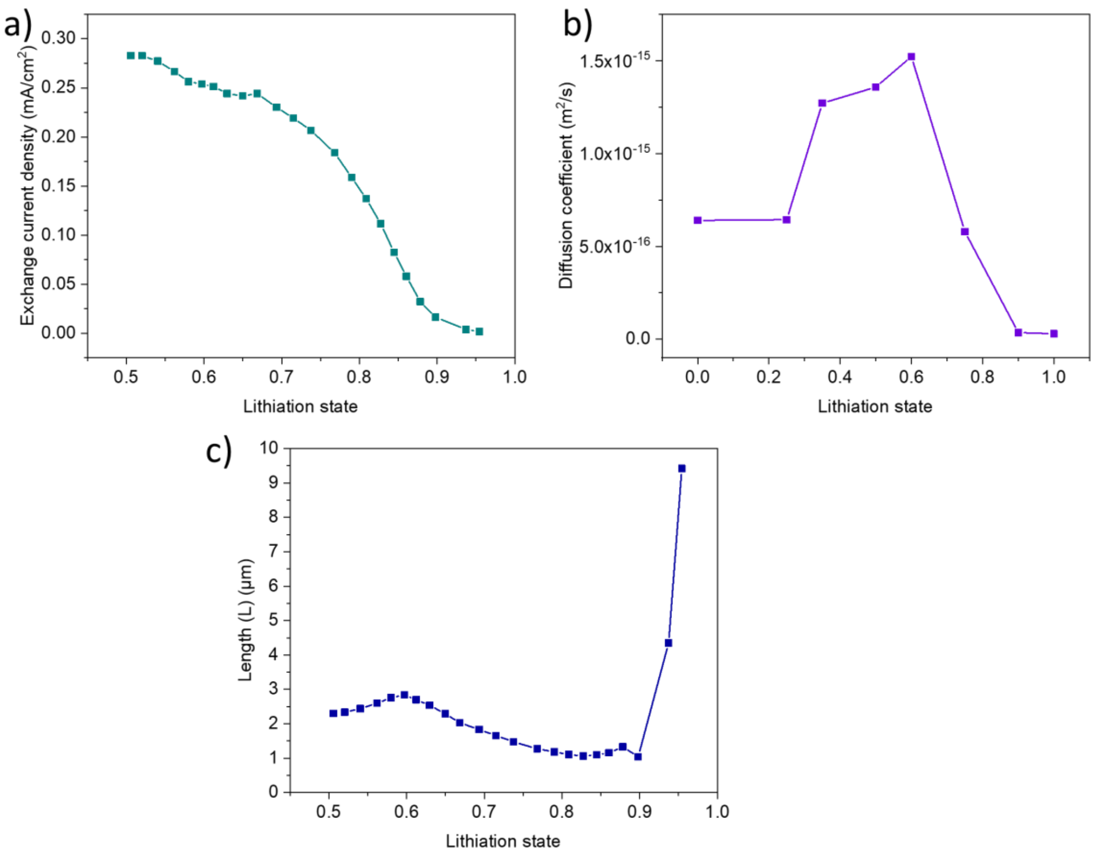


Figure S6: (a) Exchange current density values taken from ref[3] and rescaled by ref[4]. (b) The dependence of the diffusion coefficient on lithiation state, ref[2]. (c) Comparison between diffusion and surface reaction kinetics (characteristic length L in µm, which can be expressed as $D \cdot F \cdot N/j_0$).

**Note 3. Development and validation of the model**

An interesting phenomenon is the transition from kinetically-limited to diffusion-limited regimes. Results for simulations for the same pulse-rest protocol, including kinetic and/or diffusion limitations, can be observed in Figure S7. To perform those, either the parameter D or $j_0$ was kept independent of $\theta$ at its maximum value, while the other was input with its experimental dependence as explained in the methods section. A simulation with both D and $j_0$ constant at their maximum value was also performed for comparison.

Figure S7 shows that only one combination can successfully reproduce the experimental results. In particular, the experimentally found transition for the average value of $\theta$ in the basal plane from a positive slope during the first few rests, shifting to a negative one as pulses go by, can only be achieved by considering the $\theta$ dependent contribution of both D and $j_0$ parameters (Figure S7d).

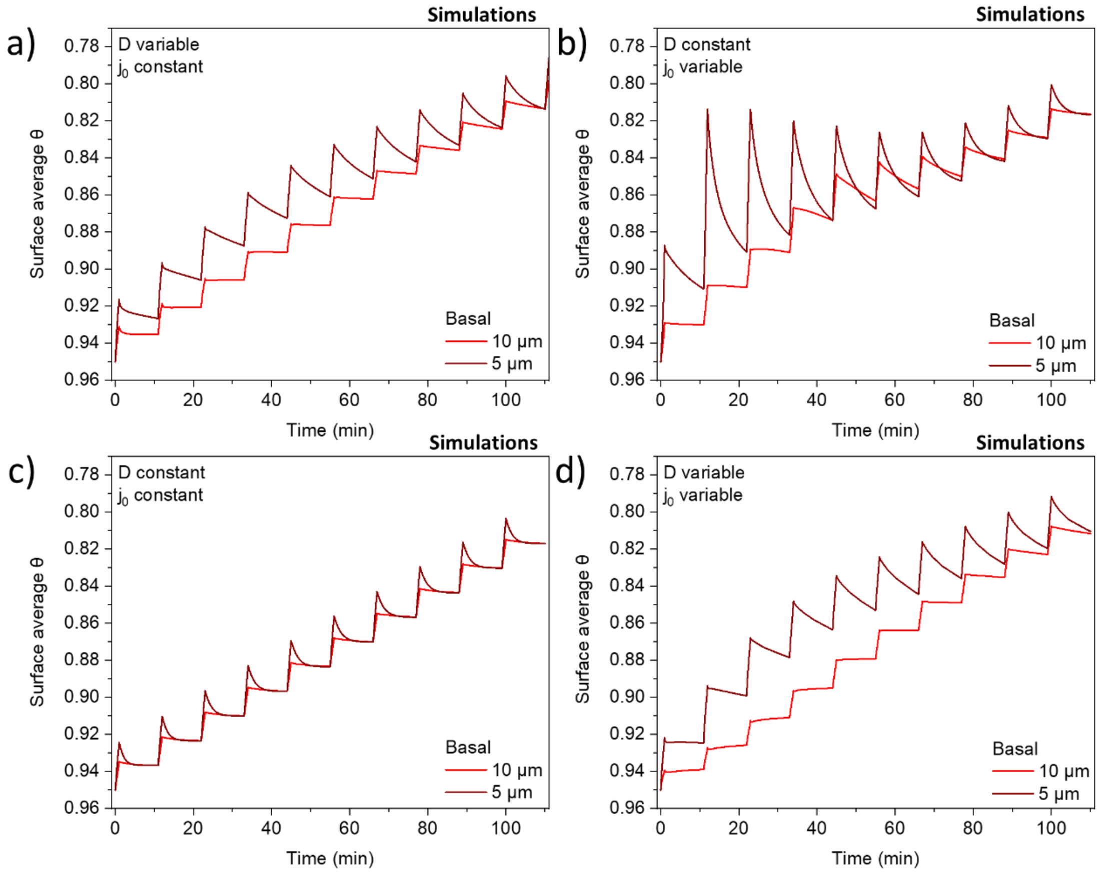


Figure S7: Simulations of 2 particles (10 and 5 µm) comparing the effects of limiting either the surface kinetics or the diffusion.

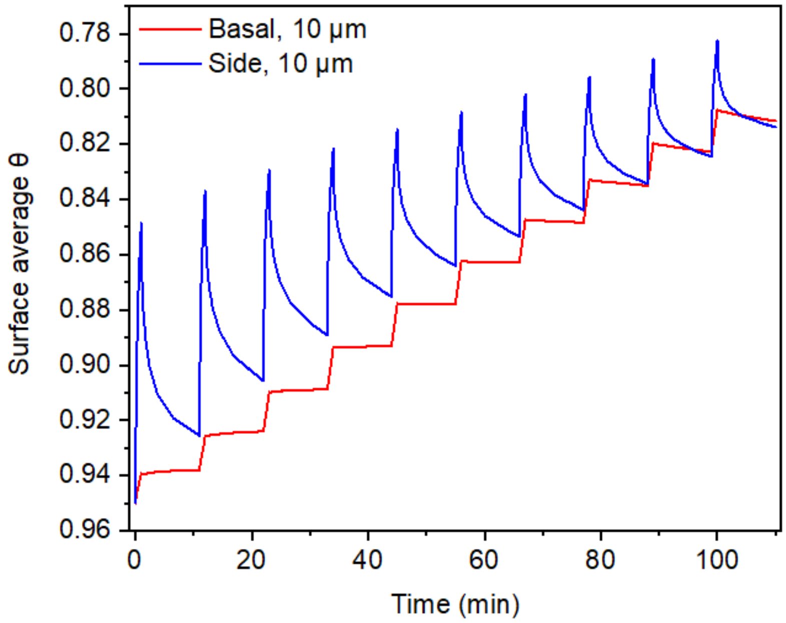


Figure S8: Results of finite element simulations showing the facet average of a lithiation (theta) state, comparing the basal and side facet of particles with 10 μm edge, for simulations comprising a 10 and 5 μm particle.

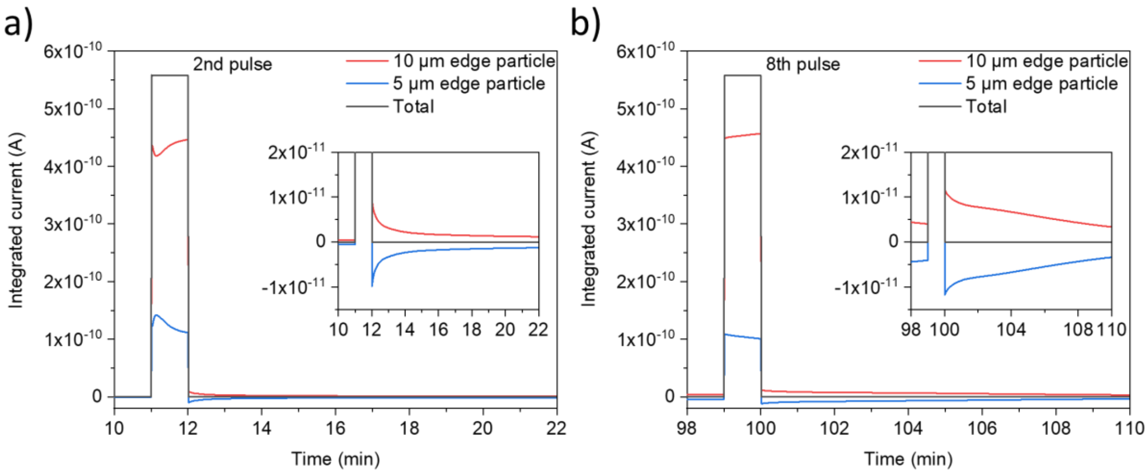


Figure S9: Total current across the side planes of each of the particles during (a) the second and (b) eighth pulse-rest periods. Insets represent a zoom into the currents during rest.

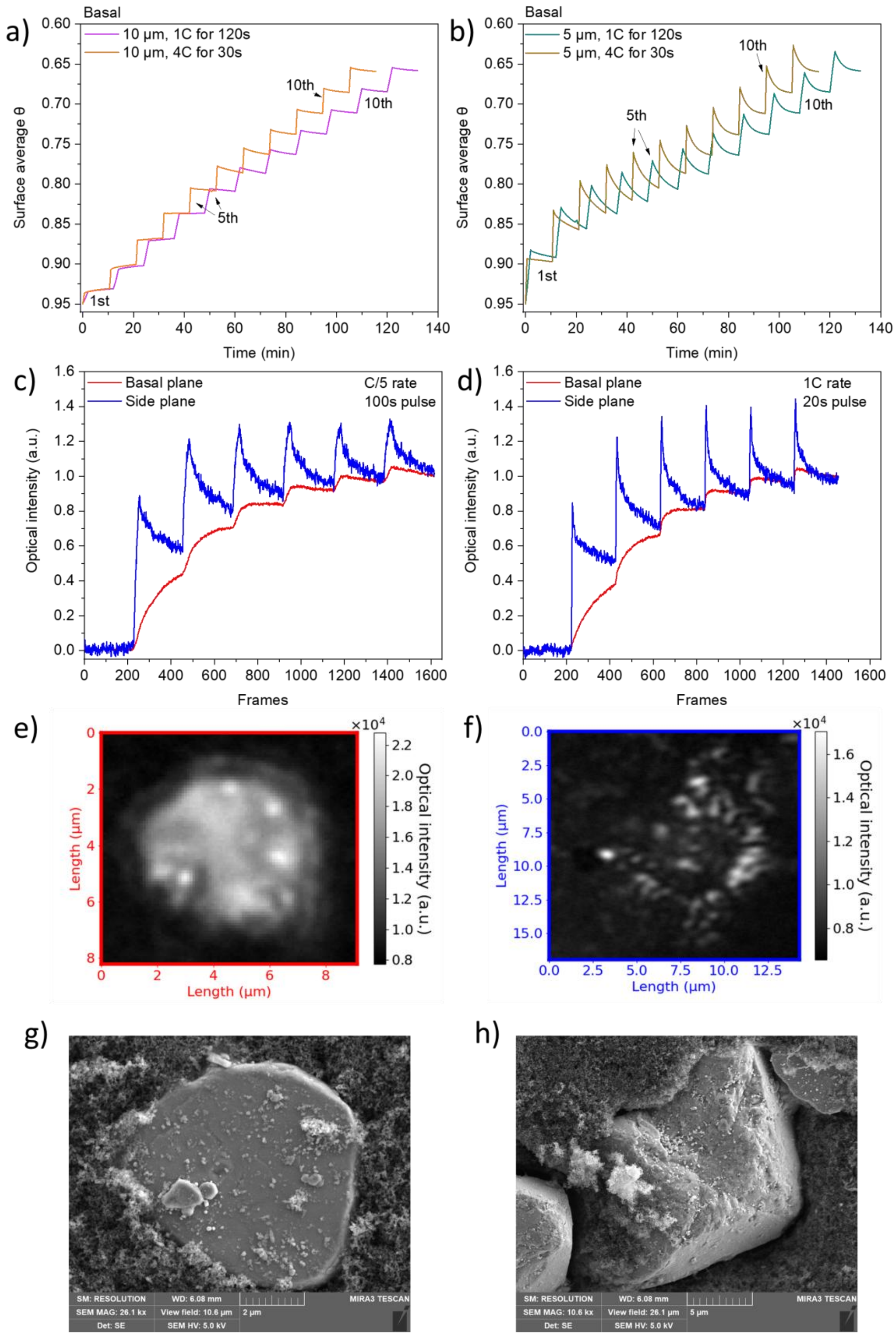


Figure S10: (a–b) Lithiation state of the basal planes for 10 and 5 μm particles obtained by the simulation of charge-rest experiments of the system composed of the two-sized octahedral particles, where the same amount of charge is passed during each pulse, but the C-rate and time are varied. When comparing the rest steps between C-rates, it is evident that the slope of variation and behaviour is

equivalent when the passed charge is the same. (c–d) Optical intensity curves representing particles oriented with basal plane and side plane up, when pulsed with C/5 for 100s and 1C for 20 s (therefore injecting the same charge), while resting for 10 minutes. The first pulse is set to 0s before starting the charge-rest experiment. A C/3 discharge to 3V, followed by a 10 min potential hold at 3 V, was performed in order to fully lithiate the particles. (e–f) Optical images of the two imaged particles, with (g–h) SEM images of the two particles.

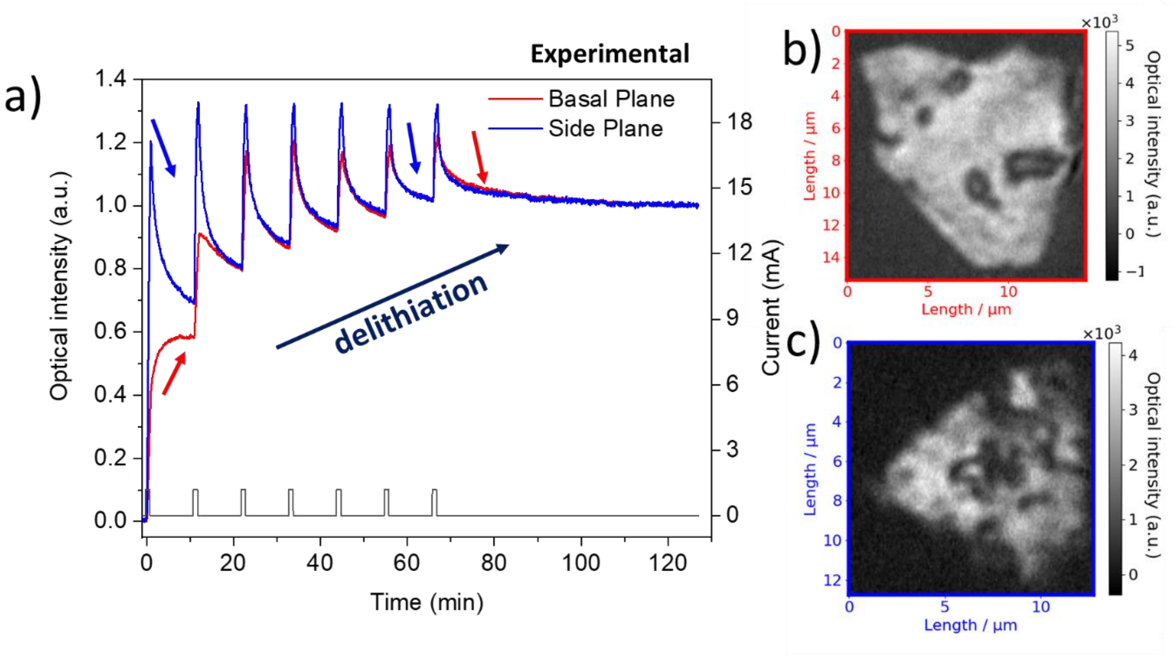


Figure S11: (a) Optical intensity curves representing particles oriented with basal plane and side plane up, when pulsed with 1C for 1 min, resting for 10 minutes and last rest for more than an hour. The first pulse is set to 0s before starting the charge-rest experiment. A C/3 discharge to 3V, followed by a 10 min potential hold at 3V, was performed in order to fully lithiate the particles. (b–c) Optical images of the two optically imaged particles from a 40 µm-thick electrode.

Table S1: Parameters (shape and scale) corresponding to the lognormal distributions represented in Figure 4a, used to generate 10 particles of random edge sizes.

| | Shape | Scale / µm |
|---|---|---|
| A | 0.093 | 7.98 |
| B (exp) | 0.31 | 7.98 |
| C | 0.465 | 7.98 |
| D | 0.62 | 7.98 |
| E | 0.31 | 2.38 |
| F | 0.093 | 2.38 |
| G | 0.155 | 3.56 |
| H | 0.31 | 3.56 |

Finally, simulations comprising 10 particles to reproduce the experimental particle size distribution were simulated. The evolution of the basal plane lithiation state can be seen in Figure S12, where the effects are similar to the previous case, which considered only two particles.

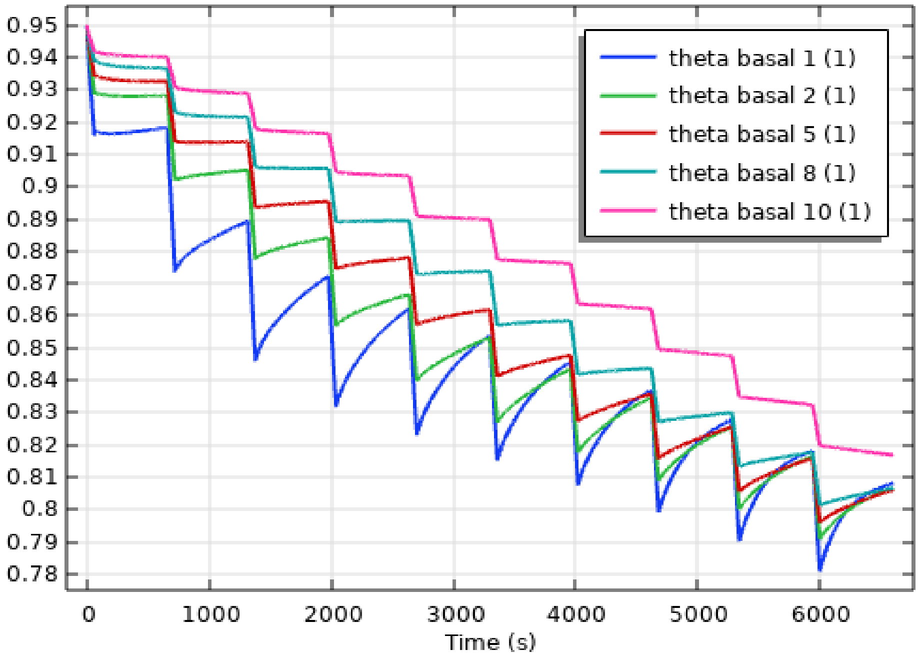


Figure S12: Selected evolution of 5 particles from a simulation of the 10 particles corresponding to the experimental B (exp) shown in Figure 6b. Evolution of the average lithiation state across the basal plane of each particle is noted in the legend.

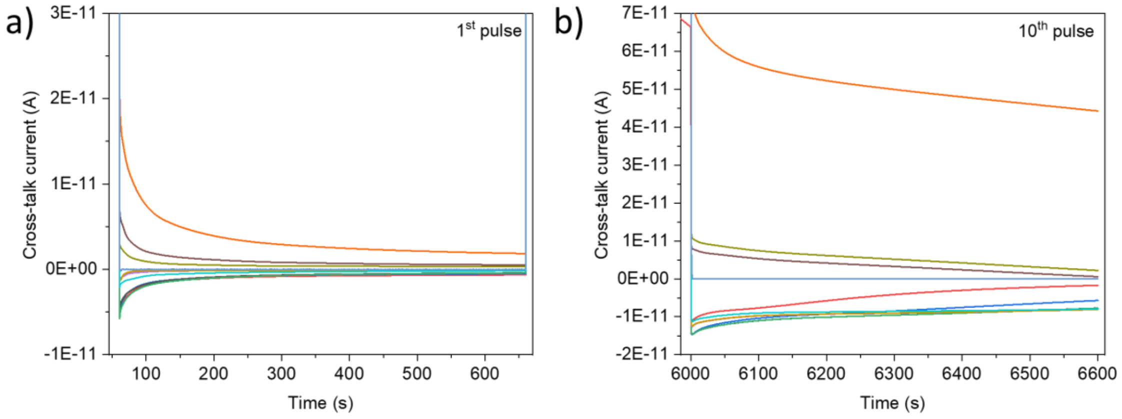


Figure S13: The evolution of a cross-talk current during a rest period for all particles in the simulation of the 10 particles corresponding to the experimental B (exp) shown in Figure 6b, (a) after the first pulse and (b) after the 10th pulse.

**References:**


(1) Xu, C.; Merryweather, A. J.; Pandurangi, S. S.; Lun, Z.; Hall, D. S.; Deshpande, V. S.; Fleck, N. A.; Schnedermann, C.; Rao, A.; Grey, C. P. Operando Visualization of Kinetically Induced Lithium Heterogeneities in Single-Particle Layered Ni-Rich Cathodes. *Joule* **2022**, *6* (11), 2535–2546. https://doi.org/10.1016/j.joule.2022.09.008.

(2) Pandurangi, S. S.; Hall, D. S.; Grey, C. P.; Deshpande, V. S.; Fleck, N. A. Chemo-Mechanical Analysis of Lithiation/Delithiation of Ni-Rich Single Crystals. *J. Electrochem. Soc.* **2023**, *170* (5), 050531. https://doi.org/10.1149/1945-7111/acd47e.

(3) Park, J.; Zhao, H.; Kang, S. D.; Lim, K.; Chen, C.-C.; Yu, Y.-S.; Braatz, R. D.; Shapiro, D. A.; Hong, J.; Toney, M. F.; Bazant, M. Z.; Chueh, W. C. Fictitious Phase Separation in Li Layered Oxides Driven by Electro-Autocatalysis. *Nat. Mater.* **2021**, *20* (7), 991–999. https://doi.org/10.1038/s41563-021-00936-1.

(4) Chen, C.-H.; Planella, F. B.; O'Regan, K.; Gastol, D.; Widanage, W. D.; Kendrick, E. Development of Experimental Techniques for Parameterization of Multi-Scale Lithium-Ion Battery Models. *J. Electrochem. Soc.* **2020**, *167* (8), 080534. https://doi.org/10.1149/1945-7111/ab9050.
